# A Phase‐Resolved Geometric Deep Learning Framework Maps Structural Determinants of Disease‐Associated Protein Aggregation and Guides Suppressor Design

**DOI:** 10.1002/advs.76118

**Published:** 2026-06-17

**Authors:** Jia Shen Sio, Wei Xuan Wilson Loo, Yan Shan Loo, Wen Xin Tan, Hui Xuan Lim, Huitao Liu, Chen Seng Ng

**Affiliations:** ^1^ School of Science Monash University Malaysia Jalan Lagoon Selatan Selangor Malaysia; ^2^ Department of Pharmaceutics and Biopharmaceutics University of Marburg Robert‐Koch‐Str. 4 Marburg Germany; ^3^ Department of Biomedical Sciences Sir Jeffrey Cheah Sunway Medical School Faculty of Medical and Life Sciences Sunway University Jalan Universiti Selangor Malaysia; ^4^ Sunway Microbiome Centre Faculty of Medical and Life Sciences Sunway University Jalan Universiti Selangor Malaysia; ^5^ Henan Linker Technology Key Laboratory College of Advanced Interdisciplinary Science and Technology Henan University of Technology Zhengzhou China

**Keywords:** artificial intelligence, computational biology, deep learning, enhancer, fibril, language model, nucleation, prnp, protein aggregation, suppressor

## Abstract

Protein aggregation drives major neurodegenerative diseases, yet most computational predictors collapse assembly into static risk scores and do not resolve the distinct structural determinants of nucleation and elongation. Here, we present SKALE 2.0, a phase‐resolved geometric deep learning framework that represents proteins as multimodal structural graphs and learns mutation‐induced aggregation phenotypes directly from three‐dimensional topology. Across SOD1, TDP‐43, MAPT, and PRNP, SKALE 2.0 recovered a conserved latent transition from nucleation to elongation while resolving distinct mutation‐specific phase sensitivities. Representative protein language model, AlphaFold‐derived feature, and non‐phase‐aware structural baselines failed to recover both phase‐dependent mutation modulation and phase separability, indicating that explicit phase conditioning is essential. The learned geometry showed that nucleation is preferentially coupled to buried hydrophobic perturbations, whereas elongation is shaped by solvent‐accessible interfaces that support fibril propagation. This framework explains how pathogenic variants can remain globally folded yet acquire aggregation competence through localized structural rewiring. Recombinant SOD1 experiments validated predicted suppressor, enhancer, and phase‐switch mutations, demonstrating that initiation and propagation can be tuned independently. SKALE 2.0 links atomic topology to phase‐specific assembly kinetics and enables a constraint‐aware design of aggregation suppressors.

## Graphical abstract

SKALE 2.0 maps disease‐associated protein aggregation as a phase‐resolved structural process, linking mutation‐induced geometric perturbations to nucleation, elongation, and suppressor design. Across neurodegenerative proteins, the framework reveals cryptic aggregation vulnerabilities, separates phase‐concordant and phase‐switching mutations, and guides sparse SOD1 suppressors that reduce aggregation while preserving native stability.

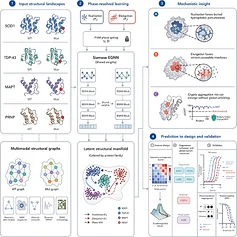

## 1 Introduction

Proteostasis depends on the reliable folding of polypeptides into native conformations that are sufficiently stable to resist off‐pathway self‐association, yet this balance is frequently disrupted in neurodegenerative diseases [1, 2]. In disorders such as amyotrophic lateral sclerosis (ALS), frontotemporal dementia, tauopathies, and prion disease, metastable proteins can traverse rugged energy landscapes and convert into highly ordered amyloid assemblies. Aggregation commonly proceeds through nucleation‐dependent polymerization, in which proteins first overcome a substantial energy barrier to form transient oligomeric nuclei, followed by rapid fibril growth once a productive template has emerged [3, 4, 5]. Although the underlying proteins differ markedly in sequence, fold, and cellular context, they share a central vulnerability in which native‐state constraints are ultimately overtaken by the thermodynamic favorability of the cross‐β amyloid state.

This principle is well illustrated across several disease‐associated systems. In ALS, superoxide dismutase 1 (SOD1) normally adopts a stable homodimeric β‐barrel architecture, yet pathogenic mutations or post‐translational perturbations can destabilize local regions and expose aggregation‐prone surfaces [6, 7, 8]. In tauopathies and frontotemporal dementia with parkinsonism, the microtubule‐associated protein tau (MAPT) forms neurotoxic filaments, often through mutations or biochemical changes that reshape its disordered conformational landscape and microtubule interactions [9, 10]. In prion disorders, the cellular prion protein undergoes template‐directed conversion into a pathogenic conformer with self‐propagating properties [11, 12]. TAR DNA‐binding protein 43 (TDP‐43) adds a further layer of complexity as an RNA‐binding protein whose pathogenic assembly reflects an interplay between structured and low‐complexity regions. Across these systems, aggregation is not simply a consequence of misfolding in the classical sense, but a kinetic and structural re‐routing of the protein into alternative assembly pathways.

A persistent paradox in this field is that disease liability often defies simple stability‐based expectations. Although global destabilization can promote aggregation, many pathogenic variants retain near‐native folds or show only modest structural perturbation while exhibiting aggressive assembly behavior in vitro or in vivo [13, 14, 15, 16]. This dissociation indicates that pathogenicity can arise from subtle topological frustration, localized geometric distortion, altered surface exposure, or rewiring of long‐range structural coupling, rather than from overt unfolding alone [13, 17]. Distinguishing these structurally subtle pathogenic variants from benign substitutions therefore remains a central challenge in protein biophysics and molecular medicine.

Computational methods have historically inferred aggregation propensity from sequence composition, motif enrichment, or static structural descriptors. Motif‐based heuristics have been valuable for identifying hydrophobic, low‐complexity, or β‐prone segments, but they often misclassify globular proteins in which these regions are buried or conformationally restrained [18, 19, 20]. Machine learning approaches improved predictive performance by integrating structure‐derived physicochemical features such as solvent accessibility, hydrogen‐bond organization, and residue environment [18, 21, 22]. Our previous framework, SKALE 1.0, showed that combining such descriptors enhances prediction relative to sequence‐only baselines [23]. However, feature‐engineering strategies still tend to represent proteins as collections of localized scalar measurements aggregated across windows or residue neighborhoods. As a result, they remain poorly suited to capture long‐range allosteric coupling, mutation‐induced redistribution of aggregation sensitivity, or the distinct structural determinants that govern nucleation and fibril growth.

Here, we instead treat aggregation propensity as a property of protein geometry that must be learned from the three‐dimensional organization of the fold itself. We developed SKALE 2.0 as a phase‐resolved geometric deep learning framework that encodes each protein as a multimodal graph derived from atomic topology and compares wild‐type (WT) and mutant states through a Siamese equivariant graph neural network (EGNN). This architecture preserves invariance to rigid‐body transformations while retaining sensitivity to local and distal geometric perturbations introduced by mutation. To resolve the temporal structure of aggregation, SKALE 2.0 incorporates learnable phase tokens that modulate latent representations through feature‐wise linear modulation (FiLM), enabling the model to separate determinants associated with nucleation from those associated with elongation within a shared structural manifold.

Using this framework across SOD1, TDP‐43, MAPT, and prion protein (PRNP), we uncover a conserved phase organization in which early aggregation is more strongly linked to perturbations within buried hydrophobic regions, whereas later growth is more strongly influenced by solvent‐accessible features that support fibril propagation. This phase‐resolved view explains how variants can remain apparently well‐folded under conventional structural proxies yet acquire pronounced aggregation competence through localized structural rewiring. We then validate these predictions experimentally in recombinant SOD1, identifying suppressor, enhancer, and phase‐switch variants that independently modulate lag time and propagation kinetics. Finally, by inverting prediction into a constrained search problem, we show that the learned aggregation landscape can be used to prioritize and design sparse suppressor mutations that reduce fibrillization while preserving native fold stability. This study positions SKALE 2.0 as a framework for linking atomic topology to phase‐specific aggregation behavior and for moving from pathogenic prediction to mechanism‐guided design.

## 2 Results

### 2.1 A Geometric Deep Learning Framework for Phase‐Resolved Kinetic Prediction

**FIGURE 1:**
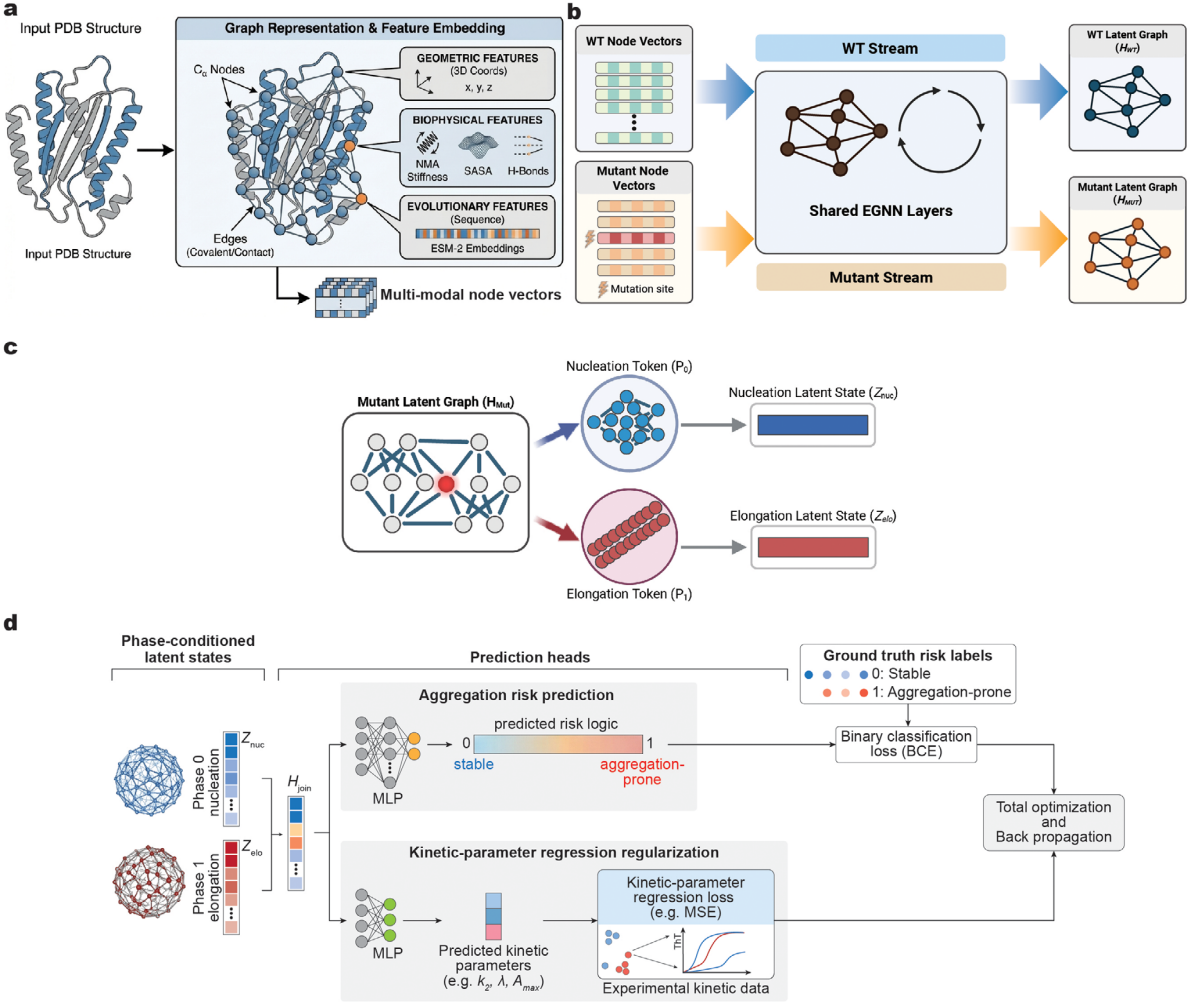
The SKALE 2.0 biophysical graph neural network framework for kinetic phase decoupling. (a) Construction of multi‐modal protein graphs where Cα nodes integrate geometric coordinates (*x*, *y*, *z*), biophysical parameters including conformational dynamics (normal mode analysis, NMA), SASA, and hydrogen bonding networks alongside evolutionary sequence embeddings. (b) Parallel processing of WT and mutant inputs via a Siamese EGNN with shared weights to generate E(3)‐equivariant node embeddings that are pooled into invariant graph‐level latent representations (*H_WT_* and *H_Mut_*). (c) Phase‐token conditioning where nucleation (*P_0_*) and elongation (*P_1_*) tokens modulate latent representations via FiLM to extract phase‐specific hidden states (*Z_nuc_* and *Z_elo_*). (d) Aggregation risk is predicted from phase‐conditioned latent representations using kinetic‐parameter regression, with binary classification applied when risk labels are available, thereby anchoring the learned geometry to experimentally relevant macroscopic aggregation behavior.

To overcome the limitations of static structural predictors in capturing the temporal organization of amyloid formation [24], we developed SKALE 2.0 as a geometric deep learning framework that resolves nucleation and elongation as distinct kinetic processes. The model represents protein conformational ensembles as multimodal graphs in which Cα nodes integrate three‐dimensional coordinates together with residue‐level biophysical features, including normal mode stiffness, solvent accessibility, hydrogen‐bond organization, and evolutionary sequence embeddings (Figure 1a). WT and mutant inputs are processed through a Siamese EGNN with shared weights, allowing both states to be compared in a common latent space that is invariant to rigid‐body transformations while remaining sensitive to mutation‐induced local geometric perturbations (Figure 1b). To resolve the kinetic phase within this latent manifold, the network incorporates learnable phase‐gating tokens implemented through FiLM [25] to selectively extract structural features associated with nucleation or elongation (Figure 1c). Aggregation risk is predicted from these phase‐conditioned representations using joint supervision from binary risk classification and kinetic‐parameter regression, which anchors the learned geometry to experimentally relevant aggregation behavior (Figure 1d). This framework provides a basis for examining how neurodegenerative mutations reshape aggregation propensity across distinct phases of assembly.

### 2.2 Latent Manifold Organization Quantifies Mutation‐Induced Structural Perturbation

**FIGURE 2:**
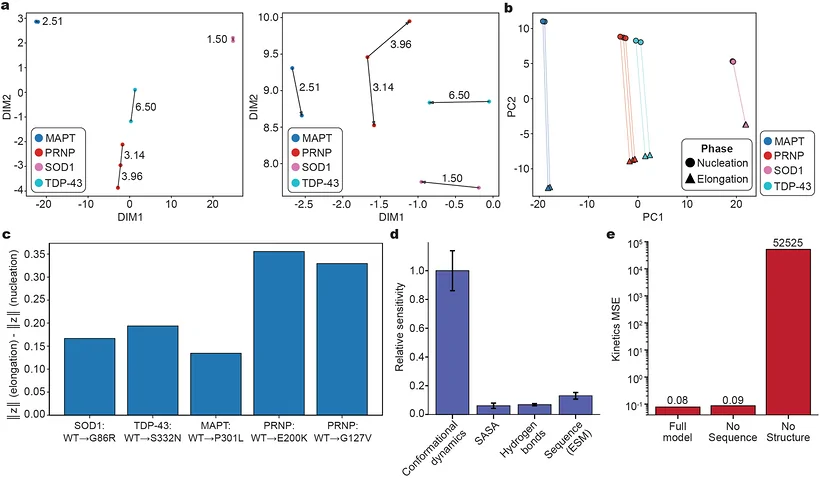
Biophysical latent space dynamics and structural interpretability. (a) Dimensionality reduction utilizing PCA (left) and UMAP (right) of learned latent embeddings for the validation cohort, colored by protein family: MAPT (blue), PRNP (red), SOD1 (pink), and TDP‐43 (cyan). Arrows trace the WT to mutant shifts, annotated by the Euclidean magnitude (∣∣Δz∣∣) computed in the latent space. (b) PCA of mean‐pooled hidden‐state embeddings *h* extracted under nucleation (*P*_0_) and elongation (*P*_1_) phase tokens. Circles denote *P*_0_ and triangles *P_1_*; trajectories connect phase states for the same variant. (c) Phase‐specific mutation impact quantified as ||Δ*z*||*_elo_* − ||Δ*z*||*_nuc_*, where ||Δ*z*||*_P_* is the WT→mutant distance computed within phase *P* (after shared standardization across phase). (d) Relative feature sensitivity of the aggregation risk logit calculated as mean absolute input gradients ||∂*R/*∂𝓍|| aggregated across structures (s.e.m.), with sensitivities normalized to the NMA channel. (e) Kinetics prediction MSE under modality ablation, where “No Sequence” masks ESM2 embeddings and “No Structure” masks coordinate geometry (*y*‐axis: logarithmic scale).

Having established the model architecture, we next examined the organization of the learned latent space by projecting validation‐set embeddings with principal component analysis (PCA) and uniform manifold approximation and projection (UMAP) (Figure 2). The manifold segregated clearly by protein family, indicating that the model first captures fold‐level structural constraints before resolving mutation‐specific effects. Within each family, WT and mutant states were connected by directed trajectories, consistent with mutation‐induced displacement in latent space. PCA showed strong global separation among MAPT, PRNP, SOD1, and TDP‐43, whereas UMAP recapitulated the same family‐resolved organization in a nonlinear embedding while preserving the WT‐to‐mutant transitions within each protein group (Figure 2a). The concordance between linear and nonlinear projections indicates that the family‐structured organization is a stable property of the learned representation rather than an artefact of a single visualization method. We quantified mutation‐induced perturbation as the Euclidean norm of the latent shift vector (‖Δ*z*‖). TDP‐43 showed the largest shift at approximately 6.50, whereas SOD1 showed a comparatively smaller shift at 1.50. MAPT occupied an intermediate range at 2.51, and the two PRNP variants showed shifts of 3.14 and 3.96 (Figure 2a). Because these values are defined in the shared latent space and are reflected consistently in both PCA and UMAP visualizations (Figure 2a), they indicate that the model does not assign a uniform family‐level penalty, but instead resolves mutation‐specific structural effects within the same protein scaffold. These results establish ‖Δ*z*‖ as a continuous measure of mutation‐induced structural perturbation across diverse amyloidogenic proteins.

We next asked whether this latent organization also resolves aggregation phase. To isolate phase‐specific structure, we projected mean‐pooled embeddings conditioned on nucleation and elongation tokens (Figure 2b). PCA of the phase‐conditioned manifold showed that the first principal component primarily separated protein families, whereas the second principal component separated nucleation states from elongation states. In each protein group, nucleation embeddings (circles) were consistently positioned above their matched elongation embeddings (triangles) along PC2, producing phase‐linked trajectories with a shared overall orientation despite differences in fold class. This conserved phase shift indicates that progression from nucleation to elongation is encoded as a common transformation in latent space rather than as a protein‐specific rearrangement.

We next assessed whether pathogenic mutations exert unequal effects across kinetic phases by comparing the difference in latent perturbation magnitude between the elongation and nucleation subspaces (Figure 2c). For every variant examined, the elongation‐associated shift exceeded the nucleation‐associated shift, yielding a uniformly positive differential. The largest differences were observed for the prion protein variants E200K and G127V, at approximately 0.35 and 0.33, respectively. More moderate shifts were observed for TDP‐43 S332N at 0.19, SOD1 G86R at 0.17, and MAPT P301L at 0.13. These results indicate that mutation‐induced structural perturbation is consistently amplified in the elongation regime relative to nucleation.

To identify the features underlying this phase dependence, we quantified the contribution of each input modality by computing the mean absolute gradient of the predicted aggregation risk with respect to each feature block (Figure 2d). Normal mode‐derived conformational dynamics showed the strongest sensitivity and served as the reference channel. By comparison, solvent accessible surface area (SASA) and hydrogen‐bond features contributed only weakly, at approximately 0.05 and 0.06 relative sensitivity, respectively, whereas evolutionary sequence embeddings contributed modestly more at approximately 0.13. These results indicate that phase‐resolved aggregation prediction is dominated by conformational dynamics, with substantially smaller contributions from static geometric descriptors and sequence‐derived embeddings. We then tested this dependence directly through systematic modality ablation during inference (Figure 2e). Masking sequence embeddings caused only minimal reduction in predictive performance, with the kinetics mean square error (MSE) increasing from 0.08 in the full model to 0.09. By contrast, masking coordinate geometry produced a catastrophic loss of accuracy, increasing the MSE to approximately 5.2 × 10^4^. These results indicate that SKALE 2.0 depends predominantly on structural geometry encoded by the folded protein, whereas sequence embeddings make only a modest additional contribution.

To place these modality‐level effects in a broader modelling context, we benchmarked SKALE 2.0 against three representative baselines. These included a protein language model baseline built from mean‐pooled evolutionary scale modeling 2 (ESM2) embeddings, an AlphaFold‐derived feature baseline built from pooled SASA, hydrogen‐bond, and normal mode channels, and a structure‐based deep learning baseline that retained the EGNN backbone but omitted phase conditioning (Figure S1). We first asked whether each model could assign different mutational consequences to nucleation and elongation by quantifying the absolute difference between WT‐to‐mutant latent displacement magnitudes across the two phases (Figure S1a). Under this criterion, all three non‐phase baselines collapsed to a phase modulation index of 0, indicating that each mutation was effectively assigned the same impact in both aggregation stages despite differences in input representation and model complexity. By contrast, full SKALE 2.0 yielded a phase modulation index of 0.948 ± 0.257 (Figure S1a), indicating a clear separation between nucleation‐linked and elongation‐linked mutational effects within the same structural pairs. This distinction was also reflected in the mean WT‐to‐mutant impact magnitudes, which remained identical across phases for the ESM2‐only, structure‐feature‐only, and EGNN‐backbone‐without‐phase‐gating baselines at 0.113 versus 0.113, 1.022 versus 1.022, and 2.384 versus 2.384, respectively, but diverged in the full model at 3.426 in nucleation and 2.478 in elongation (Figure S1c).

We next examined whether the learned representations could be separated by aggregation stage using a phase‐probe classifier applied to paired nucleation and elongation embeddings. Here again, all three non‐phase baselines remained at chance, with a phase‐probe accuracy of 0.5 (Figure S2b) and a silhouette score of zero (Figure S2c), indicating an absence of recoverable phase structure in their latent spaces. Full SKALE 2.0, in contrast, reached a phase‐probe accuracy of 1.0 with a positive silhouette score of 0.345 (Figure S2b,c), showing that the phase‐aware latent space was not only separable by classifier readout but also geometrically organized by stage. The benchmark summary sharpened this contrast by showing that only the full SKALE 2.0 architecture recovered both nonzero phase‐dependent mutation modulation and complete phase separability, whereas the language‐only, feature‐driven, and non‐phase‐aware structural baselines failed on both criteria (Figure S1c). Within the present cohort, this pattern supports the view that the principal advantage of SKALE 2.0 lies not simply in detecting structural perturbation, but in resolving how mutational effects are redistributed across distinct stages of aggregation kinetics.

### 2.3 Phase Tokens Disentangle Structural Determinants of Nucleation and Elongation

Building on the separation of kinetic phases in latent space, we next asked whether SKALE 2.0 assigns distinct structural determinants to nucleation and elongation through phase‐specific gating. We therefore examined the gradient‐based saliency maps generated under nucleation (*P*_0_) and elongation (*P*_1_) tokens, which identify the input features most strongly contributing to early and late aggregation behavior. This analysis revealed two contrasting aggregation archetypes, comprising phase‐aligned and phase‐decoupled sensitivity profiles.

**FIGURE 3:**
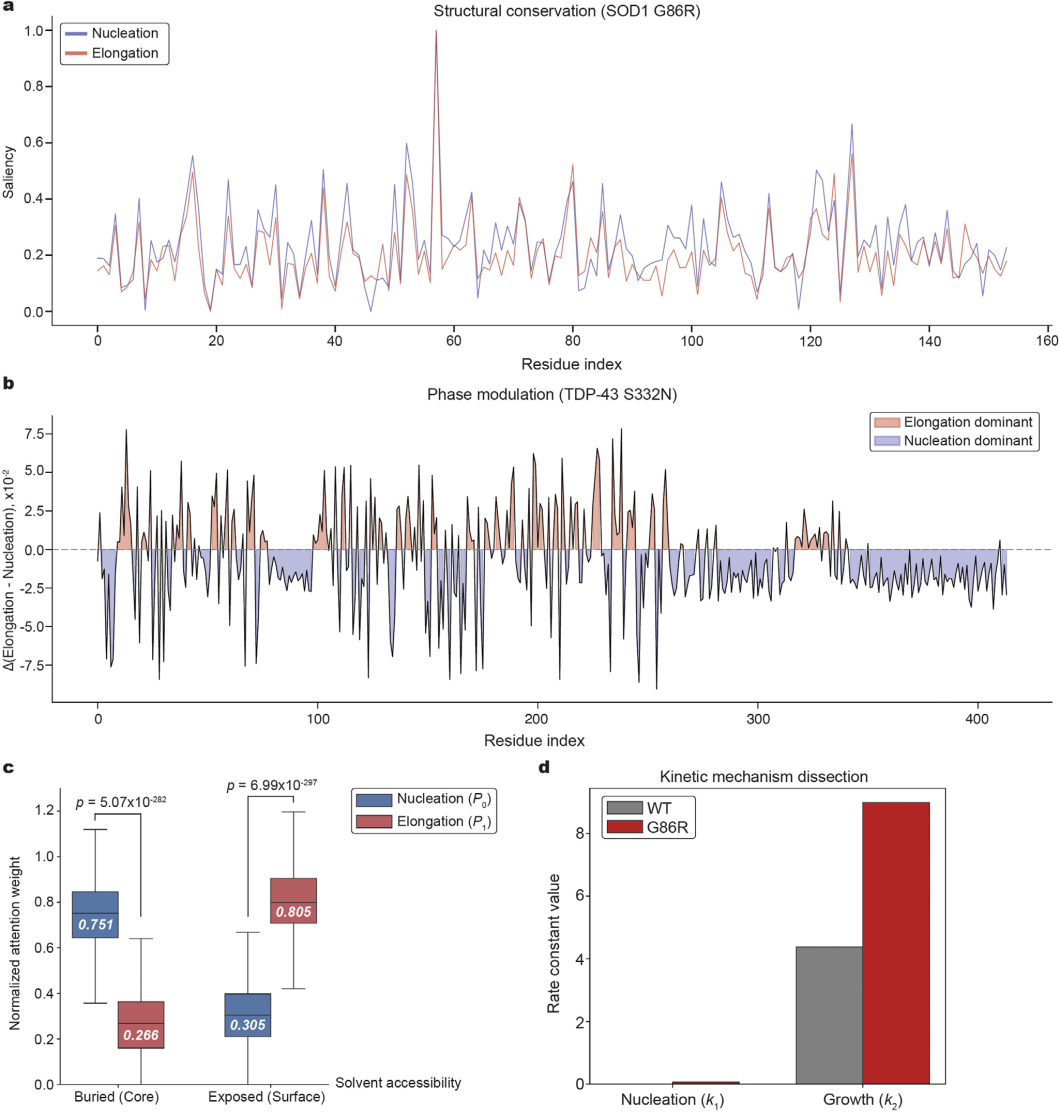
Phase‐specific structural attention and kinetic rate dissection. (a) Normalized mean gradient‐based saliency scores (∂Logit/∂𝓍) derived from phase‐specific latent conditioning for SOD1 G86R. Blue lines represent the nucleation phase token and red lines represent the elongation phase token. Saliency scores were normalized within each protein across residues for each phase token. (b) Differential phase saliency profile for TDP‐43 S332N displaying elongation‐dominant regions in red and nucleation‐dominant regions in blue calculated as the elongation score minus the nucleation score. Raw residue‐level saliency differences are shown without smoothing. (c) Global distribution of phase‐specific saliency scores stratified by solvent accessibility, representing the normalized sensitivity for nucleation (*P_0_*, blue) and elongation (*P_1_*, red) tokens assigned to buried versus solvent‐exposed residue populations. Statistical comparisons between phase tokens within each category were performed using a two‐sided Welch's *t*‐test. Center lines denote the median (values indicated inside each box); box limits indicate the 25th and 75th percentiles; whiskers extend to 1.5 times the IQR. (d) Predicted kinetic rate constants for the nucleation phase (*k_1_*) and the growth phase (*k_2_*) in arbitrary units comparing the WT baseline (grey) against the SOD1 G86R mutant (red). Rate constants *k_1_* and *k_2_* were jointly predicted from the same phase‐resolved model.

In the SOD1 G86R mutant, saliency profiles generated with nucleation (blue) and elongation (red) phase tokens were broadly concordant across the sequence, with closely aligned peaks and troughs and a shared dominant maximum near residue 58 (Figure 3a). The overall overlap indicates that much of the structural sensitivity in G86R is retained across phases, consistent with a common set of features contributing to both aggregation initiation and subsequent fibril growth. By contrast, the TDP‐43 S332N mutant displayed a phase‐decoupled saliency pattern, with alternating elongation‐dominant and nucleation‐dominant regions distributed across the sequence (Figure 3b). In this differential profile, positive excursions (red peaks) identify residues with greater elongation‐associated sensitivity, whereas negative excursions (blue troughs) identify residues with greater nucleation‐associated sensitivity. Notably, the C‐terminal portion of the sequence was biased toward negative values, indicating a broader nucleation‐dominant contribution in this region, whereas positive peaks were more punctate and localized (Figure 3b). These features indicate that the structural determinants of early and late aggregation can diverge substantially within the same mutant, supporting the existence of phase‐decoupled aggregation programs within a shared latent framework.

We next asked whether these phase‐dependent patterns reflect isolated cases or a broader property of the learned representation by stratifying saliency scores across the full validation cohort according to solvent accessibility (Figure 3c). The nucleation token (*P*_0_) showed greater sensitivity for buried core residues, with a median normalized saliency weight of 0.751 compared with 0.266 for elongation (*p* = 5.07 × 10^−282^). By contrast, the elongation token (*P*_1_) showed greater sensitivity for solvent‐exposed residues, with a median normalized saliency weight of 0.805 relative to 0.305 for *P_0_* (*p* = 6.99 × 10^−297^). These results support a phase‐dependent redistribution of structural sensitivity in which early aggregation is weighted more strongly toward buried regions, whereas later growth is weighted more strongly toward accessible surface features that support fibril recruitment.

We next examined whether these phase‐resolved structural signatures translate into distinct kinetic outputs by examining the rate constants predicted by the model (Figure 3d). In G86R, the nucleation rate constant (*k*_1_), remained low and changed only modestly relative to WT, whereas the elongation rate constant (*k*_2_), increased by more than twofold. This pattern indicates that the mutation preferentially enhances fibril growth while having comparatively minimal effect on the initiation step. These results suggest that aggregation‐promoting features in G86R become most consequential after nucleation has been established.

### 2.4 Cryptic Aggregation Risks Emerge Without Global Structural Destabilization

**FIGURE 4:**
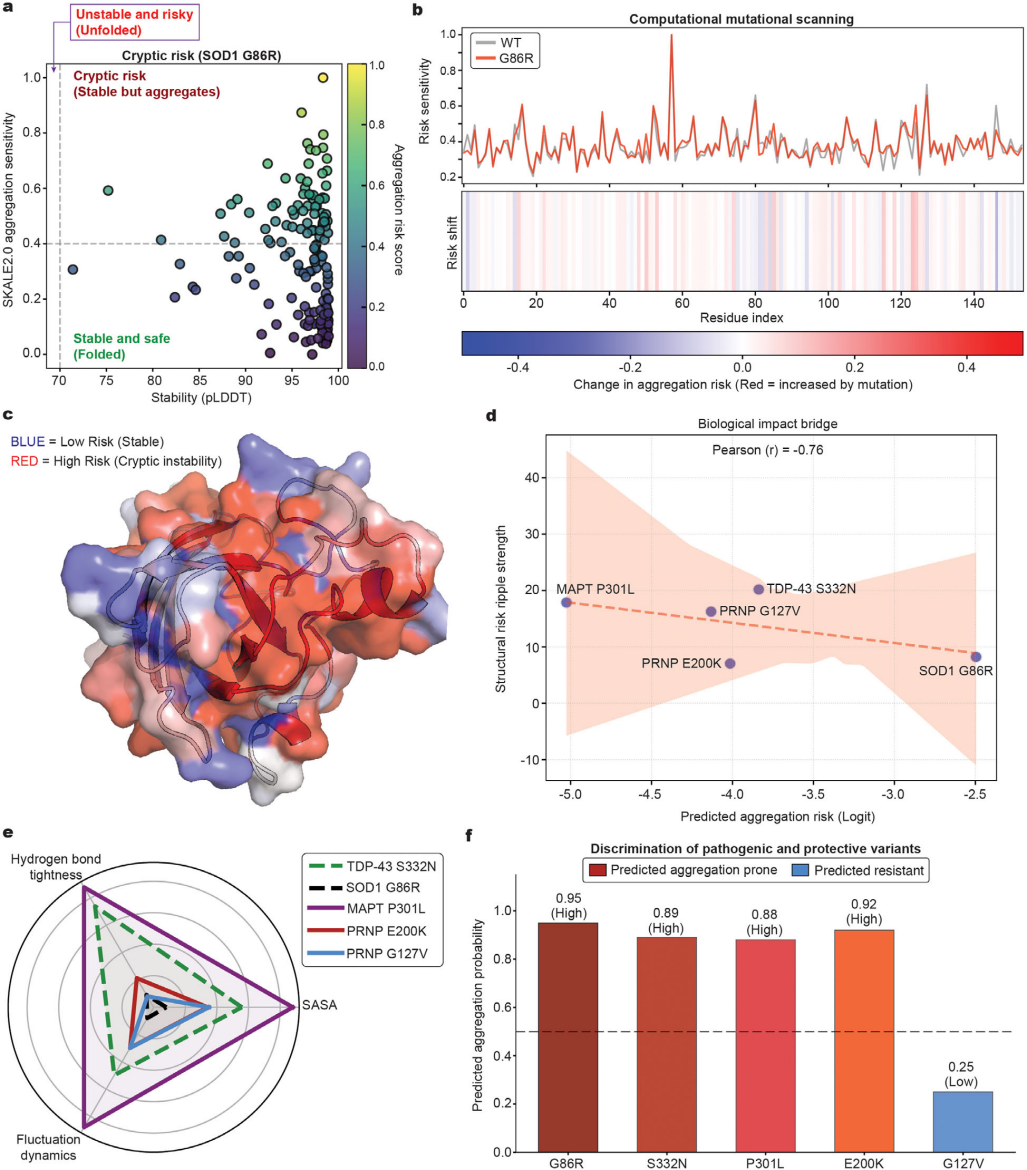
SKALE 2.0 maps cryptic aggregation risks and structural perturbation across scales. (a) Stability‐risk landscape for SOD1 G86R. Residue‐level AlphaFold confidence (pLDDT) and SKALE 2.0 aggregation sensitivity in SOD1 G86R, with risk saliency highlighting residues that remain structurally confident yet highly sensitive to aggregation‐driving perturbations. Here, pLDDT serves as a proxy for local structural confidence rather than a direct measure of thermodynamic stability. (b) Comparative risk sensitivity profiles for WT and G86R across the SOD1 sequence (top) together with a differential risk‐shift heatmap (bottom) quantifying mutation‐induced changes in aggregation sensitivity at each residue position (Red = increased risk; Blue = decreased risk). Risk shifts are reported in normalized aggregation sensitivity units. (c) 3D risk surface projection. The aggregation risk sensitivity scores (gradient norms) from panel a are mapped onto the solvent‐accessible surface of the SOD1 G86R structure. Red regions indicate predicted high sensitivity patches on the folded structure. (d) Predicted global aggregation risk and structural ripple strength across disease‐associated variants from SOD1, TDP‐43, MAPT, and PRNP, with ripple strength defined as the L1 norm of the absolute difference between normalized gradient‐norm profiles from panel b. (e) Multidimensional structural fingerprints summarizing solvent accessibility, hydrogen‐bond tightness, and atomic fluctuation, with values min–max normalized across the variant cohort. Dashed traces denote training baseline variants and the solid traces denote zero‐shot cross‐species test variants. (f) Zero‐shot aggregation probabilities produced by SKALE 2.0 across the evaluated variant set, with the dashed horizontal line marking the binary classification threshold at 0.5.

Motivated by the observation that pathogenic aggregation can occur without strong acceleration of nucleation, we next asked whether SKALE 2.0 can identify aggregation‐prone regions that are not captured by conventional structural confidence proxies. Comparing residue‐level AlphaFold confidence scores (pLDDT) with predicted aggregation sensitivity in SOD1 G86R revealed a non‐linear confidence‐risk landscape (Figure 4a). Although residues in the low‐confidence range (pLDDT < 70) tended to show elevated aggregation sensitivity, a substantial fraction of residues with high confidence (pLDDT > 85) also showed aggregation sensitivities greater than 0.6. Notably, within the strongly folded regime around pLDDT values of 97 to 99, aggregation sensitivity spanned a broad range from near baseline to almost 1.0 (Figure 4a). These data indicate that local structural confidence and latent aggregation risk are not tightly coupled, and that SKALE 2.0 can detect cryptic aggregation liability in regions that remain strongly fold‐compatible according to conventional structure predictors.

We next asked whether these vulnerabilities reflect intrinsic properties of the SOD1 scaffold or mutation‐induced effects by comparing residue‐resolved risk sensitivity profiles between WT and G86R (Figure 4b). Although the two profiles shared a broadly similar baseline pattern, the pathogenic substitution redistributed aggregation sensitivity across the structure rather than simply increasing risk at the mutation site itself. The most prominent divergence occurred near residues 57–58, where G86R showed a sharp gain in sensitivity relative to WT, together with additional localized increases at distal positions. Differential risk‐shift mapping confirmed that the mutation induces both gains and losses in aggregation sensitivity across the sequence, with positive shifts reaching approximately +0.4 at discrete sites. These results indicate that the elevated sensitivity observed in high‐confidence regions arises from mutation‐specific structural rewiring rather than from uniform destabilization or stochastic variation.

To define where these redistributed vulnerabilities localize in three dimensions, we mapped residue‐level aggregation sensitivity onto the solvent‐accessible surface of SOD1 G86R (Figure 4c). High‐sensitivity residues formed broad contiguous surface patches rather than a single focal hotspot and were distributed along exposed loops and solvent‐facing edges of the folded structure. By contrast, lower‐sensitivity regions occupied more compact and less exposed areas. This spatial organization indicates that aggregation‐permissive interfaces can emerge on the exterior of an otherwise folded protein without requiring widespread disruption of the overall scaffold.

We then examined whether this dissociation between structural disruption and aggregation risk extends across a broader variant panel by comparing predicted aggregation risk with structural ripple strength, defined as the *L*_1_ norm of the difference between mutant and WT gradient profiles (Figure 4d). Across the variants examined, the analysis revealed an inverse association (Pearson *r* = −0.76), indicating that high predicted aggregation risk can coincide with only modest global structural perturbation. SOD1 G86R exemplified this behavior, combining relatively high predicted aggregation risk at approximately −2.5 logits with a ripple strength of approximately 8 units. By contrast, MAPT P301L showed lower predicted aggregation risk at approximately −5 logits together with substantially greater structural perturbation at approximately 18 units. TDP‐43 S332N and PRNP G127V also occupied the higher‐ripple, lower‐risk region, whereas PRNP E200K combined comparatively elevated risk with limited ripple. These results indicate that aggregation propensity and global structural disruption are not obligatorily coupled.

To resolve the structural basis of this divergence, we decomposed the cross‐variant perturbation patterns into constituent biophysical features using multidimensional structural fingerprinting (Figure 4e). MAPT P301L showed the largest deviations across solvent accessibility, hydrogen‐bond tightness, and fluctuation dynamics, consistent with a broadly perturbed scaffold. TDP‐43 S332N also displayed elevated values across all three axes, whereas SOD1 G86R remained near the lower end of the range with only modest deviations. The PRNP variants occupied intermediate but distinct positions, indicating that comparable overall risk can arise from different combinations of structural change. Considered together with the residue‐level sensitivity maps, these data support a mode of aggregation risk in which localized, surface‐accessible liabilities can emerge without extensive global unfolding.

Finally, we asked whether SKALE 2.0 retains discriminative power in a zero‐shot cross‐protein setting across pathogenic and protective variants (Figure 4f). All four pathogenic variants examined, SOD1 G86R, TDP‐43 S332N, MAPT P301L, and PRNP E200K, were assigned aggregation probabilities well above the 0.5 classification threshold, with scores of 0.95, 0.89, 0.88, and 0.92, respectively. By contrast, the protective PRNP G127V variant was assigned a low aggregation probability of 0.25. These results indicate that the aggregation signal captured by SKALE 2.0 is separable from gross structural disruption and can distinguish pathogenic from protective variants without protein‐family‐specific supervision.

### 2.5 Gradient‐Guided Inverse Design Identifies Distal Suppressor Candidates

**FIGURE 5:**
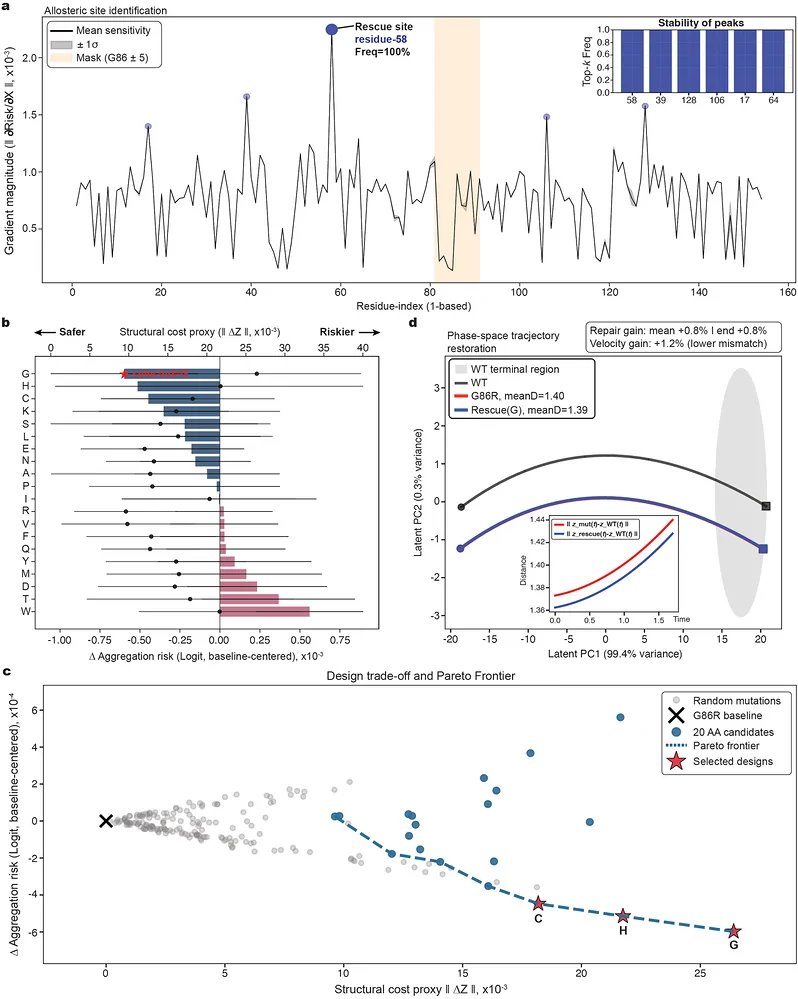
Gradient‐guided rational design of allosteric suppressors for SOD1 G86R. (a) Rescue‐site sensitivity across the SOD1 G86R sequence under the elongation phase token, quantified as the L2 norm of the gradient of the predicted aggregation‐risk logit with respect to node features (||∇_*x*_Logit||). The solid trace represents the mean across *n* = 30 noise‐perturbed replicates with Gaussian feature noise (*σ* = 0.05), and the shaded band denotes ±1 s.d. The local mutation neighborhood (G86R ± 5 residues) was masked to enrich for distal regulatory sites. The inset summarizes the frequency with which residues appeared among the top‐10 most sensitive positions across replicates, with the selected rescue site is indicated. (b) Baseline‐centered Δ*Logit* across 20 amino acid substitutions (mean ± s.d., *n *= 10 repeats), with structural cost reported as the latent displacement magnitude (||Δ*z*||). The selected design is indicated (star). Substitutions were implemented as one‐hot amino acid swaps when available, and otherwise as feature‐space perturbations; as described in Methods. (c) Structural cost (||Δ*z*||) and baseline‐centred Δ*logit* across the candidate substitutions, with the G86R reference state positioned at the origin. The random background comprises *n* = 180 single‐point mutations sampled across the protein, and the dashed line marks the non‐dominated Pareto frontier. Selected designs are highlighted and labeled. (d) Latent trajectories for WT, G86R, and the selected rescue variant generated by manual RK4 integration of the learned neural ODE from the pooled phase‐gated latent state under *P*_1_. Trajectories are projected into two dimensions by PCA, with arrows indicating temporal direction and the shaded region denoting the WT terminal region. The inset gives the time‐resolved Euclidean distance to the WT trajectory in the original latent space.

Motivated by the finding that pathogenic aggregation can arise from cryptic structural perturbations without extensive global destabilization, we next asked whether the differentiable architecture of SKALE 2.0 could be used to identify distal residues capable of modulating aggregation risk. We therefore performed gradient‐based sensitivity analysis of the SOD1 G86R mutant under the elongation phase token, while masking the local neighborhood surrounding the G86R site ( ± 5 residues), to quantify the influence of individual positions on the predicted aggregation risk score (Figure 5a). Residue 58 emerged as the dominant rescue‐site candidate, showing the highest mean sensitivity and appearing among the top ten most sensitive positions in 100% of 30 Monte Carlo noise‐perturbed replicates generated with Gaussian input noise (*σ* = 0.05). Additional recurrent peaks were observed at residues 39, 128, 106, 17, and 64; however, each showed lower mean gradient magnitudes than residue 58. These results identify residue 58 as a robust distal regulatory node for inverse design.

To assess the functional consequences of perturbing this distal site, we performed an in silico substitution scan at residue 58 and evaluated all 20 amino acid states under randomized structural perturbations (Figure 5b). Several substitutions reduced the predicted aggregation risk logit, with A58G producing the largest decrease (Δ*logit* ≈ −6 × 10^−4^), followed by A58H and A58C. In contrast, risk increased for a smaller set of substitutions, with A58W showing the strongest adverse effect (Δ*logit* ≈ +5.6 × 10^−4^). Joint comparison with latent structural cost showed that A58G achieved the greatest predicted rescue but incurred a higher displacement (‖Δz‖ ≈ 0.026) than the more conservative A58C variant (‖Δz‖ ≈ 0.018), whereas A58H occupied an intermediate regime. These results indicate that residue 58 supports a graded design landscape in which suppression efficacy and structural cost can be explicitly balanced.

To evaluate this trade‐off more systematically, we constructed a multi‐objective design landscape by plotting baseline‐centered aggregation‐risk shift against latent structural cost for all residue‐58 candidates, together with a null distribution of 180 random single‐point mutations, with the G86R baseline positioned at the origin (Figure 5c). The random mutations were concentrated near the baseline or accumulated structural cost without achieving comparable reductions in aggregation risk. By contrast, the residue‐58 substitutions traced a non‐dominated Pareto frontier, with A58C, A58H, and A58G occupying the strongest trade‐off regime. Along this frontier, progressively larger reductions in aggregation risk were accompanied by increasing structural cost, with A58G yielding the largest predicted rescue and A58C providing a more conservative alternative. This separation from the random null indicates that gradient‐guided design identifies structured suppressor candidates rather than arbitrary low‐risk perturbations.

Finally, we examined the effect of the top‐ranked A58G substitution on phase‐resolved latent dynamics by comparing neural ordinary differential equation trajectories for WT, G86R, and the rescue variant (Figure 5d). In PCA‐projected phase space, the rescue trajectory remained close to the G86R path but shifted modestly toward the WT trajectory throughout the simulated time course. Consistent with this, the rescue variant showed a small reduction in mean trajectory divergence from WT, from 1.40 for G86R to 1.39, together with improved velocity alignment and a modest gain in terminal proximity to the WT region. The inset likewise showed that the rescue trajectory remained consistently closer to WT than the G86R baseline over time. These results indicate that gradient‐guided perturbation at a distal site can partially restore the latent dynamical path learned by the model toward a less aggregation‐prone regime.

### 2.6 In Silico Saturation Mutagenesis Reveals Global and Localized Aggregation Vulnerabilities

**FIGURE 6:**
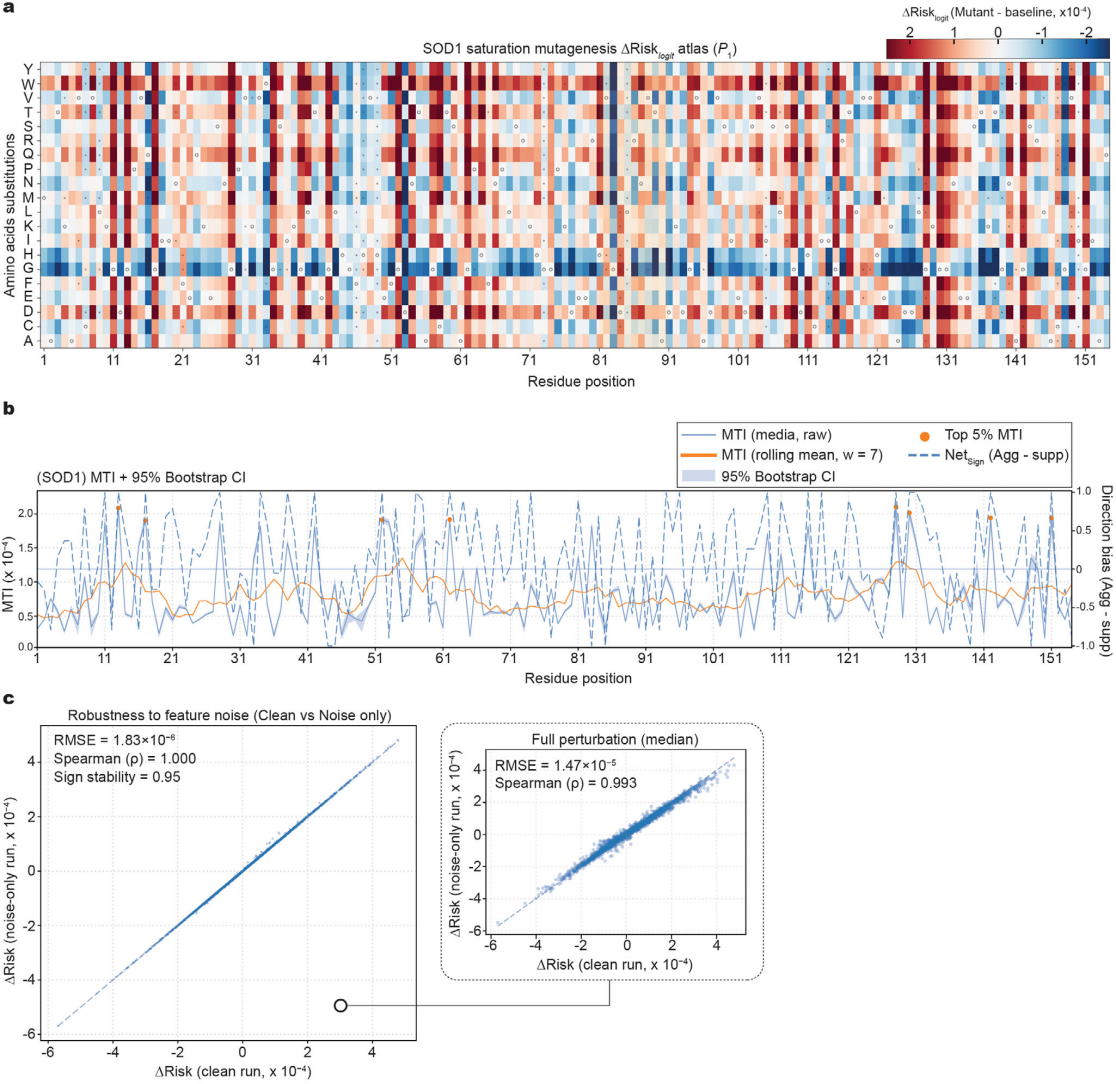
In silico saturation mutagenesis atlas with phase‐conditioned risk scoring and robustness assessment. (a) Phase‐conditioned aggregation risk shifts (Δ*Risk*_logit_) across all single amino acid substitutions and all residue positions, reported as median effects relative to WT over *K *= 30 perturbation replicates. Replicates included an unperturbed baseline, a feature‐noise condition with Gaussian noise (*σ* = 0.05), and fully perturbed conditions incorporating feature noise, coordinate jitter (*σ* = 0.4 Å), and stochastic edge dropout (*p *= 0.08). Stippling denotes high‐uncertainty entries defined by median absolute deviation in the top decile. (b) Residue‐resolved MTI, defined as the trimmed mean of absolute risk shifts (|ΔRisk_logit_|) across the 19 non‐native substitutions at each position using replicate‐median effects. Shaded bands denote 95% CI derived from block‐bootstrap resampling (*n* = 500). Dashed traces on the secondary *y*‐axis denote the fraction of substitutions at each position acting as suppressors (Δ*Risk*_logit _< 0) or aggregators (Δ*Risk*_logit _> 0). (c) Agreement between Δ*Risk*_logit_ values from the unperturbed baseline and the corresponding values obtained under Gaussian featured noise (*σ*_feat_ = 0.05) across all single amino acid substitutions, summarized by RMSE, Spearman ρ, and sign stability. The inset shows the same comparison against the median Δ*Risk*_logit_ aggregated across the full perturbation suite, including feature noise, coordinate jitter, and edge dropout, to provide a conservative robustness estimate of robustness under combined stochastic perturbation.

To extend the gradient‐guided suppressor analysis into a sequence‐wide mutational landscape, we used SKALE 2.0 to construct an in silico saturation mutagenesis atlas for the SOD1 G86R background under elongation‐phase scoring (*P*_1_), computing the predicted aggregation risk shift (Δ*Risk*_logit_) for all 2926 possible non‐native amino acid substitutions across the 154 residue positions (Figure 6a). To assess robustness, each substitution was evaluated across *K* = 30 perturbation replicates incorporating coordinate jitter (*σ* = 0.4 Å), feature noise (*σ* = 0.05), and stochastic edge dropout (*p* = 0.08), and the atlas reports the median effect. Across this perturbation regime, effects remained centered near zero, with a median shift of 9.1 × 10^−6^, a central 90% interval spanning −2.09 × 10^−4^ to +2.90 × 10^−4^, and extremes bounded between −6.6 × 10^−4^ and +6.8 × 10^−4^. Despite this overall centering, the heatmap revealed a strongly non‐uniform landscape organized into position‐specific vertical bands, indicating residues at which substitutions of diverse chemistry shifted risk in a concordant direction. A subset of positions was markedly substitution‐intolerant, with all 19 non‐native amino acid substitutions increasing predicted risk. These sites were strongly enriched at native glycine residues, including G52, G109, G115, and G128, with the largest predicted increase observed for G128W (+6.8 × 10^−4^). Uniformly risk‐reducing positions were less common, but were exemplified by W33, where all substitutions decreased predicted risk, and by D53, which produced the strongest suppression in the atlas for D53G (−6.6 × 10^−4^).

To evaluate whether substitution identity introduces a global offset that could bias interpretation of the saturation atlas, we summarized the predicted aggregation risk shift by the introduced amino acid across all residue positions (Figure S2a). This diagnostic revealed a substitution‐specific tendency in which tryptophan (W) showed the largest positive mean shift (+1.76 × 10^−4^) across 92% of positions, whereas glycine (G) showed the largest negative mean shift (−1.71 × 10^−4^) across 90% of positions; most other substitutions remained closer to zero on average. The spread across positions was nonetheless substantial, as illustrated by the cross‐sequence standard deviation for glycine (1.58 × 10^−4^), indicating that substitution‐level offsets coexist with strong position‐dependent effects. Consistent with this interpretation, the row‐effect summary statistic was 0.477, supporting a measurable substitution bias that does not fully account for the structured positional banding observed in Figure 6a. High‐uncertainty entries, defined by median absolute deviation in the top decile, were sparse and confined to a limited set of segments, indicating that the dominant hotspot and cold‐spot patterns remain stable under geometric and feature perturbations.

To distil these effects into a residue‐resolved sensitivity profile, we summarized elongation‐phase scoring using the mutational tolerance index (MTI) (Figure 6b). MTI, defined as the trimmed mean of ∣Δ*Risk*_logit_∣ across the 19 non‐native amino acid substitutions using replicate‐median effects, showed a low‐to‐moderate baseline punctuated by discrete high‐sensitivity peaks, with a median MTI of 7.5 × 10^−5^. MTI ranged from a minimum at residue 150 (1.6 × 10^−5^) to a maximum at residue 128 (3.6 × 10^−4^, 95% confidence intervals [CI] 3.48 × 10^−4^ to 3.79 × 10^−4^). The top 5% most sensitive residues comprised positions 13, 17, 52, 109, 115, 128, 130, and 142, all of which are native glycine positions. Block‐bootstrap CI (*n* = 500) were narrow across the baseline and widened preferentially at these peaks, with mean CI width increasing from 1.23 × 10^−5^ outside the top 5% to 3.32 × 10^−5^ within it, consistent with reproducible position‐level sensitivity. Because MTI captures magnitude rather than direction, we additionally quantified the net mutational sign at each residue from the balance of risk‐increasing and suppressive substitutions. Many positions supported a mixture of risk‐increasing and risk‐decreasing substitutions, whereas 20 residues behaved as strict aggregators for which every non‐native amino acid substitution increased elongation‐phase risk. By contrast, only three residues, positions 33, 83, and 118, behaved as strict suppressors for which all substitutions decreased predicted risk. Taken together, the coupled MTI and sign‐bias profiles distinguish substitution‐tolerant regions from constrained control points where single‐residue changes tend to shift elongation‐phase risk in a defined direction.

To determine whether residue‐level hotspot calls were robust to perturbation, we quantified the stability of the top 5% hotspot set across stochastic replicates by ranking residues by absolute predicted risk shift and comparing each perturbed run with the unperturbed reference (Figure S3a). Using *k* = 8 residues, the Jaccard overlap was a median of 0.455, with an interquartile range (IQR) of 0.333 to 0.455, corresponding to approximately four to five shared residues in a typical replicate. A one‐sided permutation test against a size‐matched random‐selection null confirmed that this overlap exceeded chance expectation (*p* < 0.001, *N* = 1000 permutations). We then localized this stability by computing the residue‐level inclusion frequency within the top‐5% hotspot set across perturbation replicates (Figure S3b). This analysis resolved a compact consensus core led by residue 53 (1), with recurrent inclusion of residues 16 (0.833), 58 (0.800), 128 (0.800), and 52 (0.533), whereas most positions showed near‐zero inclusion. The mutation‐centered window surrounding the G86R site (± 5 residues) served as contextual reference and was rarely recovered under this ranking criterion, with inclusion frequencies of zero at most positions and only minor inclusion at residues 81 (0.167) and 89 (0.067). These results indicate that the most sensitive hotspots are reproducibly retained across perturbation regimes and concentrate within a small, stable subset of positions rather than dispersing broadly across the sequence.

To place the substitution atlas in a quantitative robustness context, we paired the predicted aggregation risk shifts (Δ*Risk*_logit_) from the unperturbed baseline run with those obtained under feature noise alone across all single‐amino acid substitutions (*σ* = 0.05) (Figure 6c). The paired effects demonstrated an almost perfectly preserved rank and scale relationship, with a Spearman *ρ* of 1, a slope of 1.001, and an intercept of 2.48 × 10^−7^. This baseline yielded low overall variance, with a mean absolute error of 8.60 × 10^−7^ and a root mean square error (RMSE) of 1.83 × 10^−6^, while maintaining a sign stability of 0.95. Extending beyond this isolated noise condition, the replicate‐level robustness analysis showed that agreement with the unperturbed atlas was preserved across the broader perturbation suite, with a median effect‐stability Spearman *ρ* of 0.976 (IQR 0.971 to 0.981) and a median sign‐stability of 0.951 (IQR 0.945 to 0.957). A more conservative comparison against the median risk shift aggregated across the full perturbation suite likewise preserved strong agreement with the unperturbed run, yielding a Spearman *ρ* of 0.993 and an RMSE of 1.47 × 10^−5^ (Figure 6c, inset). Together with the top‐k stability analysis in Figure S3, these results indicate that the dominant substitution‐level effects and residue‐level sensitivity patterns in Figure 6a,b remain stable under both isolated feature noise and combined stochastic perturbations.

### 2.7 Generalized Mutational Landscapes and Structural Vulnerabilities Across Diverse Amyloidogenic Scaffolds

To assess generalization beyond SOD1, we constructed phase‐conditioned saturation atlases for TDP‐43 S332N, MAPT P301L, and PRNP E200K. Row‐effect diagnostics revealed measurable, protein‐specific substitution offsets, with summary statistics of 0.55 for TDP‐43 (Figure S2b), 0.609 for MAPT (Figure S2c), and 0.572 for PRNP (Figure S2d). Across all three scaffolds, tryptophan consistently produced the largest positive mean shifts, whereas glycine produced the largest negative mean shifts (Figure S2b–d). Specifically, tryptophan and glycine yielded respective mean shifts of +4.1 × 10^−5^ and −4.4 × 10^−5^ in TDP‐43, +3.4 × 10^−5^ and −3 × 10^−5^ in MAPT, and +8.51 × 10^−5^ and −6.99 × 10^−5^ in PRNP. These dominant axes were accompanied by shared secondary chemical tendencies, including positive biases for aspartate and glutamine (Gln, Q) and negative biases for histidine (His, H) across the three proteins. The substantial standard deviation whiskers in all panels indicate that these sequence‐averaged substitution biases coexist with strong position‐dependent variability rather than fully explaining the underlying atlas structure.

To isolate localized structural sensitivity, we analyzed each saturation atlas after subtracting these substitution‐specific mean shifts for each introduced amino acid (Figure S4). Under this row‐centering normalization, all three proteins retained clear position‐specific banding, indicating that residue context, rather than substitution identity alone, remains the dominant determinant of risk shifts. The resulting effect scales were protein‐specific. In TDP‐43, the central 90% range spanned −3.86 × 10^−5^ to +4.20 × 10^−5^, with the largest localized effects reaching 1.26 × 10^−4^ at residue 37 and 1.20 × 10^−4^ at residue 241 (Figure S4a). MAPT showed a narrower distribution, with the central 90% spanning −2.47 × 10^−5^ to +3.41 × 10^−5^ and peaking at 5.75 × 10^−5^ at residue 326 (Figure S4b). Conversely, PRNP demonstrated the broadest sensitivity landscape, with the central 90% spanning −7.27 × 10^−5^ to +7.27 × 10^−5^ and a maximum effect of 2.75 × 10^−4^ at residue 158 (Figure S4c). These results show that the framework resolves protein‐specific, position‐dependent aggregation landscapes with distinct effect magnitudes across diverse amyloidogenic scaffolds.

To distil these sequence‐wide substitution effects into residue‐resolved sensitivity profiles, we summarized the elongation‐phase scoring in the TDP‐43 S332N background using the MTI (Figure S5a). The MTI showed a low‐to‐moderate baseline with a median sensitivity of 1.77 × 10^−5^. Positional sensitivity ranged from a minimum at residue 150 (4.47 × 10^−6^) to a maximum at residue 37 (9.47 × 10^−5^, 95% CI 9.19 × 10^−5^ to 9.77 × 10^−5^). The top 5% most sensitive subset comprised 21 residues, including positions 37, 24, 146, and 53. Block‐bootstrap CI remained narrow across the baseline, with a mean width of 1.34 × 10^−6^, and widened preferentially at the sensitivity peaks to a mean width of 6.10 × 10^−6^. Directional analysis of the net mutational sign further identified 55 residues acting as strict aggregators, for which all non‐native amino acid substitutions increased predicted risk, whereas only 6 residues functioned as strict suppressors.

A parallel analysis of the MAPT P301L scaffold demonstrated a more constrained mutational sensitivity profile, with a median mutational tolerance index of 1.25 × 10^−5^ (Figure S5b). The tolerance profile ranged from a minimum at residue 441 (8.38 × 10^−6^) to a maximum at residue 326 (4.66 × 10^−5^, 95% CI 4.57 × 10^−5^ to 4.72 × 10^−5^). The highest sensitivity echelon comprised 23 residues, led by positions 326, 323, and 261. Consistent with the restricted dynamic range of this tau variant, the CI were exceptionally tight, averaging 4.35 × 10^−7^ outside the top 5% and expanding only modestly to 1.15 × 10^−6^ within the peaks. Directional profiling identified 49 strict aggregators and no strict suppressors, indicating a landscape strongly biased toward mutation‐induced risk exacerbation.

Evaluation of the PRNP E200K landscape revealed a higher baseline MTI and a substantially broader dynamic range than observed for other scaffolds (Figure S5c). The median MTI was 3.52 × 10^−5^, with the minimum localized to residue 209 (1.12 × 10^−5^) and the maximum reaching 1.50 × 10^−4^ at residue 131 (95% CI 1.44 × 10^−4^ to 1.62 × 10^−4^). The most vulnerable 5% of the sequence consisted of 13 structural hotspots, including positions 131, 127, and 5. Variance scaled with this elevated sensitivity, with mean CI widths of 2.98 × 10^−6^ across the baseline widening to 1 × 10^−^^5^ at the peaks. Net sign profiling identified 45 strict aggregators together with 10 strict suppressors. These coupled mutational tolerance and sign‐bias profiles define a broader and more bidirectional mutational landscape in PRNP, delineating rigid control points alongside substitution‐tolerant regions across distinct amyloidogenic folds.

To quantify replicate robustness of the substitution atlases under elongation‐phase scoring, we compared clean predicted risk shifts against a feature‐noise replicate and against the median atlas aggregated across the full perturbation suite for each target. For TDP‐43, agreement between the clean and noise‐only atlases was essentially preserved, with *ρ* = 1, RMSE = 5.88 × 10^−7^, and sign stability of 0.974 (Figure S5d), while concordance with the full‐perturbation median remained high at *ρ* = 0.995 with RMSE = 3.15 × 10^−6^ (Figure S5d, inset). For MAPT, clean versus noise‐only agreement showed similarly high precision, with *ρ* = 0.999 and RMSE = 7.43 × 10^−7^, together with a median effect‐stability *ρ* of 0.998 (IQR 0.9983 to 0.9984) and a median sign stability of 0.995 (IQR 0.994 to 0.996) (Figure S5e). Agreement with the full‐perturbation median also remained strong, with *ρ* = 0.999 and RMSE = 1.06 × 10^−6^ (Figure S5e, inset). For PRNP, clean versus noise‐only concordance was likewise strong, with *ρ* = 1 and RMSE = 1.52 × 10^−6^, accompanied by a median effect stability *ρ* of 0.989 (IQR 0.9887 to 0.9908) and a median sign stability of 0.975 (IQR 0.972 to 0.977) (Figure S5f). Concordance with the full‐perturbation median remained robust, with *ρ* = 0.996 and RMSE = 6.77 × 10^−6^ (Figure S5f, inset). Top‐residue set stability under perturbation provided a complementary residue‐ranking view, with Jaccard medians of 0.750 (IQR 0.750 to 0.826) in TDP‐43, 0.533 (IQR 0.484 to 0.586) in MAPT, and 0.625 (IQR 0.625 to 0.733) in PRNP, confirming that structural vulnerabilities are reproducibly recovered despite geometric and feature noise.

### 2.8 Local Motif Grammar Reveals Conserved Hotspots and Sparse Phase Divergence

**FIGURE 7:**
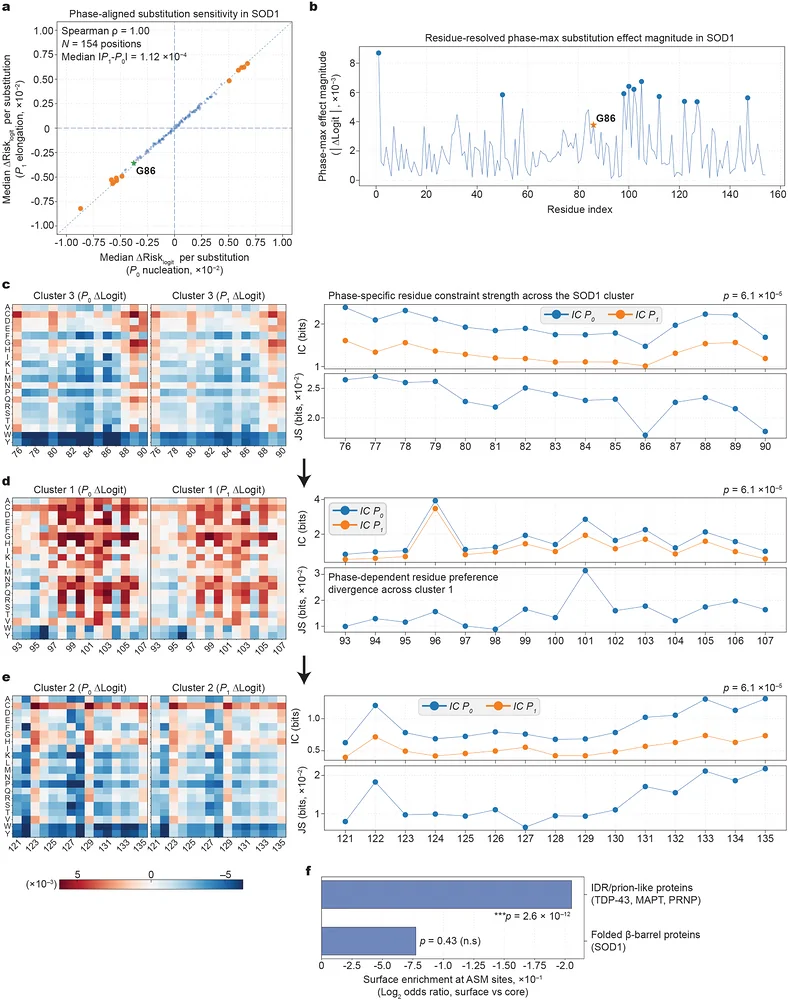
Phase‐aligned substitution sensitivity and architecture‐dependent structural context of aggregation‐sensitive residues. (a) Median Δ*Risk*_logit_ per true amino acid substitutions across WT SOD1 under nucleation (*P*_0_) and elongation (*P*_1_) phase using ESM2 re‐embedding, with dashed lines marking zero effect in each phase. Spearman correlation, residue count, and summary statistics are indicated. (b) Residue‐resolved phase‐maximum substitution effect magnitude across SOD1, defined for each residue as max(|*E_P0_* |, |*E_P1_* |) from the median Δ*Risk*_logit_ across phases. Selected hotspot residues are highlighted. (c–e) Cluster‐resolved substitution maps for three structurally distinct motifs comprising the zinc‐binding loop (c, Cluster 3), the amyloidogenic G93 core (d, Cluster 1), and the electrostatic loop (e, Cluster 2). Phase‐specific heatmaps give residue‐by‐amino acid Δ*logit* substitution effects under *P*_0_ and *P*_1_ scoring. Accompanying profiles show per‐position IC (bits) derived from softmax‐normalized substitution preference distributions for each phase, together with paired Wilcoxon *P‐*values. Phase divergence at each position is quantified using JS divergence (bits), with permutation‐derived significance markers where applicable. (f) Log_2_ OR comparing solvent‐exposed surface and buried core enrichment of aggregation‐sensitive mutation sites in folded β‐barrel proteins (SOD1) and intrinsically disordered/prion‐like proteins (TDP‐43, MAPT, PRNP), with exact *p* values from Fisher's exact test. Heatmaps color scales denote median Δ*logit* substitution effects (×10^−3^). All analyses were performed on WT structures using true amino acid substitutions evaluated by ESM2 re‐embedding.

Having established that SKALE 2.0 resolves robust, protein‐specific mutational landscapes across diverse amyloidogenic folds, we next investigated how these substitution effects are organized across kinetic phases and local structural context within the foundational SOD1 scaffold. To determine whether mutational sensitivities diverge between amyloidogenic stages, we evaluated the sequence‐wide substitution landscape of WT SOD1 across the nucleation (*P*_0_) and elongation (*P*_1_) phases. Using phase‐conditioned scoring with per‐substitution median predicted aggregation risk shifts (Δ*Risk*_logit_) across all 154 residue positions, we observed near‐complete phase concordance at the sequence level (Figure 7a). Paired phase effects preserved both rank and magnitude, with Spearman *ρ* = 0.9998 (*p* = 5.01 × 10^−259^) and Pearson *r* = 0.9999 (*p* = 6.57 × 10^−296^). Absolute phase divergence remained small, with a median |*P*_1_  −  *P*_0_| of 1.12 × 10^−4^ and a maximum of 2.04 × 10^−3^. Effect direction was preserved at 152 residues, with the only two sign inversions occurring at positions 115 and 140, where baseline effects were close to zero. Elongation effects were modestly attenuated relative to nucleation, yielding a lower median |Δlogit| in *P*_1_ (2.94 × 10^−3^) than in *P*_0_ (3.47 × 10^−3^). Consistent with this overall concordance, permutation testing with false‐discovery‐rate (FDR) correction detected no significant phase switchers. Notably, the ALS‐linked G86 site retained a suppressive profile in both phases, with median risk shifts of −8.69 × 10^−3^ in *P*_0_ and −7.25 × 10^−3^ in *P*_1_.

We then asked where these globally conserved sensitivities are concentrated along the sequence. To localize the strongest shared liabilities, we quantified the maximum absolute median risk shift across both phases for each residue, $\max(|E_{P_{0}}\left|,\;\right|E_{P_{1}}|)$ (Figure 7b). This revealed a sparse hotspot architecture superimposed on a broadly modest baseline. The median phase‐maximum magnitude was 3.47 × 10^−3^ with an IQR of 1.37 × 10^−3^ to 5.49 × 10^−3^, whereas 14 residues exceeded 1 × 10^−2^. The largest perturbations localized to discrete structural nodes, including W33, M1, E50, I100, I105, L127, and C147, together with concentrated hotspot bands spanning residues 83 to 84 and 98 to 105. The ALS‐linked G86 site lay within the upper tail of this distribution, ranking sixteenth overall with a phase‐maximum effect of 8.69 × 10^−3^. These results indicate that the sequence determinants governing SOD1 fibrillization are dominated by shared structural bottlenecks that affect both nucleation and elongation, with only limited phase‐specific divergence at the global level.

We next asked whether this broad phase concordance masks finer motif‐level differences in local substitution grammar. Cluster‐resolved substitution maps across five structurally defined SOD1 segments showed that phase effects remain largely conserved at the pattern level, while residue‐level constraint is consistently stronger during nucleation than elongation (Figure 7c–e; Figure S6a,b). In every cluster, the nucleation (*P*_0_) and elongation (*P*_1_) substitution heatmaps shared identical dominant stripes and hotspot structures (Figure 7c–e, left, and Figure S6a,b, left). However, the corresponding information‐content (IC) profiles cleanly separated the two phases, with nucleation IC remaining strictly above elongation IC throughout the evaluated windows (paired Wilcoxon *p* = 6.1 × 10^−5^) (Figure 7c–e, right, and Figure S6a,b, right). This pattern indicates that the model preserves largely identical residue‐preference ordering across phases while imposing tighter substitution selectivity during nucleation, consistent with a stronger early sequence gate in the aggregation trajectory.

Within the zinc‐binding loop spanning residues 76 to 90, the substitution landscape formed a broad central suppressive basin across both phases, together with locally enhancing effects toward the carboxyl‐terminal boundary (Figure 7c, left). Overall constraint remained high, with nucleation IC ranging from 2 to 2.6 bits and elongation IC ranging from 1 to 1.6 bits. This baseline was interrupted by a shared sequence trough at residue 86 followed by a rebound across residues 87 to 89 (Figure 7c, right). Phase divergence was modest and highly structured, with Jensen–Shannon (JS) divergence generally spanning 2.2 × 10^−2^ to 2.7 × 10^−2^, punctuated by a sharp minimum near 1.7 × 10^−2^ at residue 86 before recovering downstream (Figure 7c, right). This profile supports a globally constrained loop architecture containing a narrow permissive locus where phase preference collapses into a common profile.

Analysis of the amyloidogenic core from residues 93 to 107 revealed dense, highly structured substitution effects organized around a dominant constraint peak centered at residue 96 (Figure 7d, left). Nucleation IC rose sharply to approximately 3.8 to 4 bits at this locus, with the elongation IC reaching 3.3 to 3.6 bits, whereas surrounding positions stabilized near 0.8 to 2.8 bits in nucleation and 0.7 to 2 bits in elongation (Figure 7d, right). Phase divergence remained tightly localized, hovering largely between 1 × 10^−2^ and 2 × 10^−2^ but interrupted by a dominant divergence peak at residue 101 reaching nearly 3 × 10^−2^ (Figure 7d, right). The separation between the maximal constraint site at residue 96 and the maximal divergence site at residue 101 suggests a local division between residues enforcing strong sequence selectivity and neighboring residues mediating phase differentiation. Within the electrostatic loop spanning residues 121 to 135, overall constraint was lower and more gradually distributed, consistent with a relatively permissive solvent‐facing segment that tightened toward its distal boundary (Figure 7e, left). Nucleation IC ranged from approximately 0.6 to 1.35 bits, whereas elongation IC ranged from 0.35 to 0.75 bits. The spatial profile showed an early peak near residue 122, a flatter mid‐window regime across residues 123 to 129, and a late rise from residue 130 culminating in the highest regional constraint near residue 135 (Figure 7e, right). Phase divergence followed a matched architectural pattern, with an early JS divergence peak near residue 122 at approximately 1.8 × 10^−2^, a pronounced trough at residue 127 near 0.6 × 10^−2^, and a steady increase to a late maximum approaching 2.2 × 10^−2^ near residue 135 (Figure 7e, right). The coincident rise of both metrics at the carboxy‐terminal boundary indicates this region concentrates both thermodynamic constraint and phase discrimination.

Two N‐terminal segments detailed in the supplemental analysis extended these principles while highlighting discrete sites with permutation‐supported phase divergence (Figure S6a,b, right). In Cluster 4, spanning residues 26 to 40, structural constraint showed a bipartite organization. Nucleation IC rose from roughly 0.7 to 1.4 bits across residues 26 to 29, reached a primary maximum near residue 30 at 2.4 to 2.6 bits, formed a secondary peak near residue 34 at 2.3 to 2.5 bits, dropped to a regional minimum near residues 38 to 39 at 0.6 to 0.9 bits, and rebounded at residue 40 (Figure S6a, right). JS divergence followed the identical spatial logic, with a dominant peak near residue 30 at 2.3 × 10^−2^ to 2.5 × 10^−2^, a secondary elevation near residue 36 at 2 × 10^−2^ to 2.2 × 10^−2^, a trough across residues 37 to 38 at 0.9 × 10^−2^ to 1 × 10^−3^, and a late rise at residue 40 reaching 2 × 10^−2^ within a permutation‐significant window (*p* < 0.05). In Cluster 5, spanning residues 43 to 57, nucleation IC ranged from 0.7 to 2.2 bits and elongation IC ranged from 0.35 to 1.5 bits, with high‐constraint peaks near residues 44 and 46 at approximately 2 bits and a late peak near residue 56 at 2.1 to 2.3 bits, separated by a local minimum near residue 53 at 1 bit (Figure S6b, right). Phase divergence remained localized, anchored by a dominant peak near residue 44 at 3 × 10^−2^, sustained divergence across the central window at 1.8 × 10^−2^ to 2.5 × 10^−2^, a dip near residue 53 at 1.5 × 10^−2^, and renewed permutation‐significant elevation across residues 54 to 57 at 2.3 × 10^−2^ to 2.5 × 10^−2^ (*p* < 0.05). These supplemental segments confirm that phase divergence concentrates into a sparse subset of residues, coupling strong constraint to measurable phase‐specific preference shifts. These local maps define a motif‐specific biophysical grammar in which nucleation uniformly imposes tighter substitution selectivity than elongation across all evaluated clusters (Figure 7c–e; Figure S6a,b), while phase divergence remains sparse and heavily residue‐localized. The most prominent divergence nodes include residue 86 in the zinc‐binding loop, residue 101 in the amyloid core, the distal boundary of the electrostatic loop, and discrete amino‐terminal sites exhibiting statistically rigorous divergence.

Extending beyond local sequence windows, the spatial deployment of high‐sensitivity aggregation‐shifting sites depended heavily on protein architecture when stratified by solvent accessibility on WT structures (Figure 7f). For the folded β‐barrel protein SOD1, sensitive sites demonstrated no detectable enrichment on solvent‐exposed residues relative to buried core positions, yielding a near‐zero surface versus core log_2_ odds ratio (OR) of approximately −0.08 (Fisher exact test *p* = 0.43). By contrast, intrinsically disordered or prion‐like proteins pooled across TDP‐43, MAPT, and PRNP showed a pronounced accessibility bias, with a negative log_2_ OR of approximately −0.20 and a robust deviation from neutrality (Fisher exact test *p* = 2.6 × 10^−12^). This cross‐class comparison indicates that folded β‐barrel architectures support a relatively even surface‐to‐core distribution of mutational sensitivity, whereas disordered or prion‐like architectures concentrate sensitivity away from solvent‐exposed positions, directly linking local substitution effects to higher‐order structural context. Consistent with this architecture‐level accessibility dependence, a pooled solvent‐accessibility analysis supported a conserved geometric bias in the placement of aggregation‐shifting mutation sites (ASM) across the cohort (Figure S7). Kernel density estimates of SASA showed that sensitive sites (*N* = 149) were modestly shifted toward lower exposure than the background (*N* = 1113), with median SASA near 105 Å^2^ versus approximately 115 Å^2^, while retaining substantial overlap across the full exposure range. This global consensus indicates that solvent exposure modulates ASM likelihood without deterministically partitioning vulnerable sites, consistent with thermodynamic vulnerability arising from both partially buried packing constraints and surface‐coupled interaction interfaces.

### 2.9 Experimental Validation Confirms Phase‐Resolved Kinetic Predictions

To determine whether the phase‐resolved SKALE 2.0 computational landscape yields actionable kinetic phenotypes, we quantified the in vitro aggregation trajectories of three algorithmically nominated substitutions alongside WT SOD1 and the ALS‐linked G93A control (Figure S8). The A96Y substitution was selected as a phase‐concordant suppressor predicted to attenuate both nucleation (Δ$\mathrm{logi}t_{P_{0}}$ = −8.95 × 10^−3^) and elongation (Δ$\mathrm{logi}t_{P_{1}}$ = −9.15 × 10^−3^). The I105N variant served as a phase‐concordant enhancer predicted to accelerate both kinetic stages (*Δ* $\mathrm{logi}t_{P_{0}}$ = +7.24 × 10^−3^, $\Delta \mathrm{logi}t_{P_{1}}$ = +7.51 × 10^−3^). Crucially, K4N was selected as a decoupled phase‐switch candidate possessing opposing mutational signs (*Δ* $\mathrm{logi}t_{P_{0}}$ = −3.90 × 10^−5^, *Δ* $\mathrm{logi}t_{P_{1}}$= +7 × 10^−5^), predicting a prolonged lag phase paired with accelerated fibril growth. Recombinant proteins were expressed, purified, and assayed at scale, with 80 replicate traces per variant used to resolve both mean kinetics and inter‐replicate dispersion.

The experimental ThT trajectories separated these computational classes into distinct kinetic regimes (Figure S8a). WT SOD1 exhibited a delayed and low‐amplitude rise, establishing a baseline lag phase of 14.3 ± 5.7 h, a shallow maximal slope of approximately 6 a.u. per hour, and a terminal amplitude of 51 ± 15 a.u. In contrast, the I105N and G93A variants accelerated aggregation through early onset and steep signal growth. The engineered dual‐phase enhancer I105N shortened the lag phase to 10.1 ± 1.9 h (*p* = 3.90 × 10^−9^) and increased the apparent elongation rate to 1.45 ± 1 h^−^^1^ (*p* = 1.29 × 10^−14^), reaching half‐maximal signal at 11.5 h with a maximal slope of 44 a.u. per hour. This acceleration exceeded the G93A disease control, which displayed a 12.2 ± 4.2 h lag phase, a 13.3 h half‐maximal time, and a maximal slope of 37 a.u. per hour. Both rapidly aggregating variants peaked near 240 to 260 a.u. between 18 and 22 h.

Conversely, the dual‐phase suppressor A96Y substantially delayed and attenuated fibrillization relative to the high‐aggregation variants. The mutation extended the lag phase to 22.1 ± 4.1 h (*p* = 1.78 × 10^−18^) with a later onset near 19.9 h, slowed the elongation velocity to 0.28 ± 0.06 h^−^^1^ (*p* = 1.85 × 10^−^^1^ ^3^), reduced the maximal slope to 9 a.u. per hour, and yielded a substantially lower terminal amplitude of 128 ± 31 a.u. Strikingly, the computationally identified phase switcher K4N reproduced the predicted decoupled biophysical phenotype. The variant heavily suppressed initial seed formation, remaining near baseline until 21.2 h and extending the calculated lag phase to 24.6 ± 4.1 h (*p* = 1.65 × 10^−27^). However, upon overcoming this initial thermodynamic barrier and reaching half‐maximal signal at 26.8 h, K4N drove rapid fibril growth with an enhanced elongation rate of 0.85 ± 0.20 h^−^^1^ (*p* = 1.42 × 10^−23^) and a maximal slope of 22 a.u. per hour, ultimately reaching a high terminal amplitude of 261 ± 72 a.u (Figure S8a). These experimental readouts recapitulate the directional classes encoded by the phase‐resolved predictions, validating the ability of the SKALE 2.0 framework to separate rapidly aggregating, delayed lower‐amplitude, and lag‐extending phase‐decoupled classes within a standardized kinetic assay.

Sigmoidal fitting of individual trajectories across 80 replicates per variant provided parameter‐level validation for the SKALE 2.0 phase‐resolved kinetic classes (Figure S8b–d). Lag time, used here to quantify the effective nucleation barrier, separated the suppressive and enhancing mutational regimes (Figure S8b). Relative to the WT baseline of 14.87 ± 0.56 h, the SKALE 2.0‐predicted A96Y and K4N variants prolonged aggregation initiation to 22.08 ± 0.46 h (*p* = 3.53 × 10^−18^) and 24.63 ± 0.46 h (*p* = 1.08 × 10^−27^). Conversely, the computationally nominated I105N variant shortened this timescale to 10.11 ± 0.22 h (*p* = 3.65 × 10^−12^), with a lag time shorter than the disease‐associated G93A positive control (11.85 ± 0.25 h, *p* = 3.48 × 10^−6^). Quantification of the apparent propagation rate resolved phase‐specific modulation of fibril growth (Figure S8c). The WT scaffold propagated at 0.5 ± 0.021 h^−^^1^, whereas A96Y reduced the elongation rate to 0.277 ± 0.007 h^−^^1^ (*p* = 1.83 × 10^−16^). Consistent with kinetic decoupling predicted by SKALE 2.0, K4N increased the propagation rate to 0.853 ± 0.022 h^−^^1^ (*p* = 2.08 × 10^−22^) despite its prolonged nucleation phase. The fastest variants generated the highest *k*_app_ values, with I105N reaching 1.384 ± 0.055 h^−^^1^ (*p* = 9.27 × 10^−28^) compared with 1.120 ± 0.023 h^−^^1^ for G93A (*p* = 1.09 × 10^−44^).

Plateau intensities further separated low‐amplitude from high‐amplitude outcomes (Figure S8d). WT SOD1 plateaued at 59.12 ± 1.70 a.u., whereas A96Y moderately increased this intensity to 130.90 ± 3.57 a.u. (*p* = 2.70 × 10^−35^). Beyond this intermediate phenotype, the SKALE 2.0‐predicted K4N and I105N variants converged with the G93A control on high amyloid burdens, reaching similar high‐yield plateaus of 275.18 ± 8.32 (*p* = 1.12 × 10^−41^), 271.23 ± 13.32 (*p* = 1.51 × 10^−26^), and 269.69 ± 10.53 a.u. (*p* = 4.15 × 10^−33^), respectively. These extracted biophysical parameters are consistent with the SKALE 2.0 phase‐resolved sequence predictions. A96Y shifted the kinetic profile toward delayed initiation and reduced propagation, I105N accelerated both processes beyond known disease benchmarks, and K4N effectively decoupled the kinetic phases by extending the lag time while simultaneously elevating the propagation rate (*k*_app_) and terminal plateau (Figure S8b–d).

Normalizing each fitted kinetic parameter to the WT mean highlighted the effect sizes underlying the absolute differences (Figure S8e–g). The SKALE 2.0‐nominated A96Y suppressor shifted kinetics toward slower overall progression, increasing lag time by 48.5% ± 3.1% (Figure S8e, *p* = 3.53 × 10^−18^) and reducing the apparent propagation rate by 44.6% ± 1.3% (Figure S8f, *p* = 1.83 × 10^−16^), while increasing plateau ThT intensity by 121.4% ± 6% (Figure S8g, *p* = 2.70 × 10^−35^). Demonstrating a strictly decoupled trajectory, K4N produced the strongest initiation delay by extending the lag time by 65.6% ± 3.1% (*p* = 1.08 × 10^−27^) but simultaneously increased propagation by 70.8% ± 4.5% (*p* = 2.08 × 10^−22^) and elevated the plateau intensity by 365.5% ± 14.1% (*p* = 1.12 × 10^−41^). The rapid aggregators exhibited coordinated shifts toward accelerated nucleation and enhanced propagation (Figure S8e–g). The disease‐linked G93A control shortened lag time by 20.3% ± 1.7% (*p* = 3.48 × 10^−6^), increased propagation by 124.1% ± 4.6% (*p* = 1.09 × 10^−44^), and elevated the plateau intensity by 356.2% ± 17.8% (*p* = 4.15 × 10^−33^). I105N further shortened the lag time by 32% ± 1.5% (*p* = 3.65 × 10^−12^) and accelerated propagation by 176.9% ± 10.9% (*p* = 9.27 × 10^−28^), while reaching a similarly high plateau intensity increase of 358.8% ± 22.5% (*p* = 1.51 × 10^−26^). Collectively, these normalized macroscopic comparisons confirm that SKALE 2.0 single‐residue phase signatures directly translate to precision‐engineered shifts in lag, propagation, and plateau amplitude, effectively separating initiation from growth within a single sequence scaffold.

### 2.10 Generative Design Reveals a Sparse Structural Network Governing Aggregation Suppression

**FIGURE 8:**
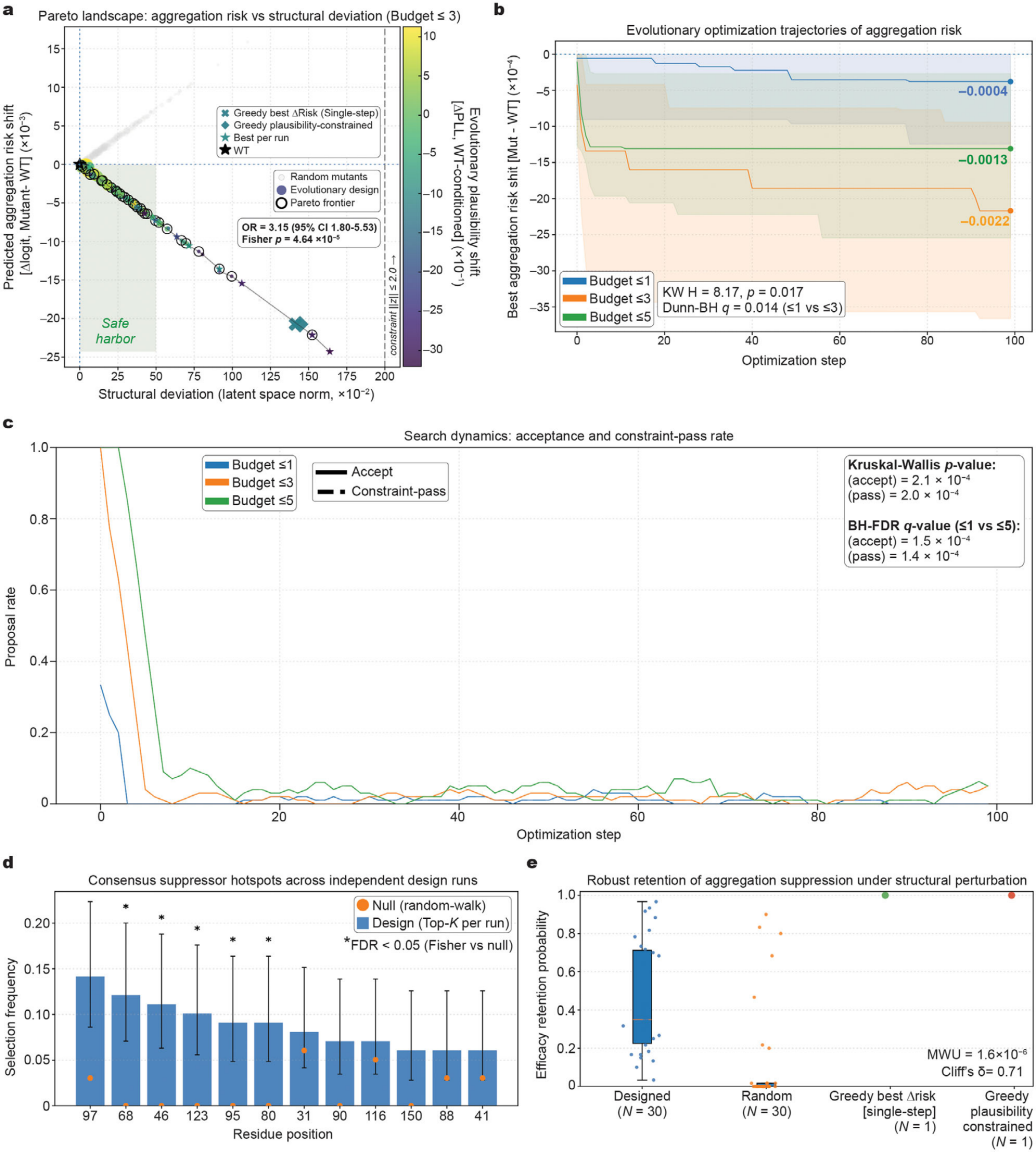
Multi‐objective evolutionary design, search dynamics, and robustness of aggregation suppressors. (a) Aggregation‐risk shift relative to WT (∆logit) and latent structural deviation (∥Δz∥) across sequences generated under a mutation budget of ≤3, shown against a background of random mutants. Open black circles mark the Pareto frontier, greedy single‐step and plausibility‐constrained baselines are shown for comparison. The shaded region denotes the predefined safe harbor regime, and the color scale encodes the WT‐conditioned protein language model plausibility shift (∆*PLL*). Inset statistics give the OR and two‐sided Fisher's exact *p*‐value for safe‐harbor enrichment relative to random variants. (b) Median best‐so‐far aggregation risk shift across 100 optimization steps under mutation budgets of ≤1, ≤3, and ≤5, with shaded IQR ranges spanning *N *= 20 independent runs per budget. The dotted horizontal line marks the WT baseline. Final step differences were assessed by Kruskal–Wallis testing with Dunn's post‐hoc correction and Benjamini‐Hochberg adjustment. (c) Rolling means (window size = 5) of Metropolis proposal acceptance rates (solid lines) and constraint‐pass rates (dashed lines) across optimization time for each mutation budget. (d) Residue‐level selection frequency across top‐performing designs is shown (blue bars) with 95% Wilson CI. Orange dots indicate the null recurrence expectation from a budget‐matched random‐walk sampling. Asterisks denote significant enrichment over the null expectation by one‐sided Fisher's exact test with BH‐FDR < 0.05. (e) Efficiency‐retention probabilities under structural perturbation across evolutionary designs (*N *= 30) and random controls (*N *= 30), with single‐sequence greedy baselines shown for reference. Retention was defined as the fraction of perturbation realizations preserving a negative Δ*Risk*. Statistical comparison between designed and random variants was performed using a two‐sided Mann–Whitney *U*‐test with Cliff's δ effect size.

We next asked whether the SKALE 2.0 predictive framework could be inverted to design aggregation suppressors rather than simply predict their effects. Having established that SKALE 2.0 accurately predicts single‐residue kinetic phenotypes in vitro, we repurposed the learned latent representation as a generative design engine to identify multistep aggregation suppressors. Candidate sequences were explored using multi‐objective evolutionary search constrained to a maximum of three substitutions. Candidate sequences were evaluated within a joint design landscape defined by predicted aggregation suppression (ΔRisk), latent structural displacement (‖Δ*z*‖), and evolutionary plausibility measured by WT‐conditioned protein language model log‐likelihood shifts (Δ*PLL*) (Figure 8a). Whereas 400 randomly sampled mutational combinations formed a dense manifold containing 142 risk‐increasing variants, the evolutionary search recovered a compact and enriched set of 70 suppressor designs. The resulting Pareto frontier revealed a graded biophysical coupling in which deeper predicted suppression was associated with progressively larger structural displacement.

To prioritize fold‐preserving variants and maintain sequence plausibility, we defined a stringent safe‐harbor regime restricting structural deviation to ‖Δ*z*‖ ≤ 0.5 for suppressor mutations (Δ*Risk *< 0). The evolutionary algorithm concentrated optimized variants within this structurally permissive region, placing 51 of the 70 generated designs inside the safe harbor compared with 184 of the 400 random background mutants. This enrichment corresponded to an OR of 3.15 (95% CI 1.80 to 5.53, Fisher exact *p* = 4.64 × 10^−5^) (Figure 8a). Consistent with constraint‐aware generation, these safe‐harbor designs exhibited improved evolutionary plausibility relative to the random controls, shifting the median Δ*PLL* from −0.48 to −0.32.

Mapping the extremes of the design landscape highlighted the importance of multi‐objective optimization. The strongest suppressor reached a Δ*Risk* of −2.43 × 10^−2^ but fell well outside the safe harbor, incurring a large structural displacement (‖Δ*z*‖ = 1.64) and a substantial plausibility penalty (Δ*PLL* = −4.15). In contrast, the best safe‐harbor design balanced these competing constraints, achieving a Δ*Risk* of −7.29 × 10^−3^ with a minimal structural deviation of 0.496 latent units and a modest plausibility shift of −1.77. Greedy single‐step baselines achieved similarly deep suppression (Δ*Risk* ≈ −2.07 × 10^−2^) but failed to enter the safe harbor due to large structural displacement (‖Δ*z*‖ ≈ 1.43) (Figure 8a). These results indicate that SKALE 2.0 can identify mutational trajectories that reduce aggregation propensity while preserving sequence plausibility and structural integrity.

To evaluate the efficiency and scaling limits of this generative search, we tracked aggregation risk trajectories across 100 optimization steps under mutational budgets allowing ≤1, ≤3, and ≤5 substitutions (Figure 8b). Across 20 independent runs per budget, the search produced rapid early reductions in predicted aggregation risk before stabilizing into distinct performance plateaus. Allowing up to three substitutions produced the deepest and most consistent improvements, reaching a final median ΔRisk shift of −2.17 × 10^−3^ with an IQR from −3.66 × 10^−3^ to −9.43 × 10^−4^. This configuration established an early advantage, achieving a median shift of −1.34 × 10^−3^ by step 2 and continuing to refine sequences through later iterations. Restricting the search to single substitutions yielded weaker and less consistent improvements. The ≤1‐substitution budget reached a final median shift of −3.79 × 10^−4^ with an IQR from −1.25 × 10^−3^ to 2.68 × 10^−7^, indicating that a subset of runs failed to identify suppressors within the optimization horizon. Expanding the search to a ≤5‐substitution budget did not improve the median outcome, plateauing at −1.31 × 10^−3^ with an IQR from −2.54 × 10^−3^ to −2.69 × 10^−4^. These results indicate that the ≤3 budget provided the most effective balance between search flexibility and constraint satisfaction, whereas the ≤1‐substitution budget was too restrictive and the ≤5‐substitution budget expanded combinatorial freedom without improving the final median outcome. Global comparison of final optimized states revealed significant differences among mutational budgets (Kruskal–Wallis *H* = 8.17, *p* = 0.0169). Post hoc comparisons with Benjamini–Hochberg correction showed that the ≤3‐substitution budget significantly outperformed the single‐mutation baseline (*q* = 0.014), whereas extending the search to five substitutions produced no significant improvement. These results indicate that aggregation suppression can be achieved through a sparse and coordinated mutational network rather than widespread sequence modification.

To investigate the mechanistic basis of these optimization plateaus, we analyzed rolling five‐step averages of Metropolis proposal acceptance and constraint‐pass rates across the search trajectory (Figure 8c). Acceptance probabilities declined sharply after departure from the WT sequence and subsequently stabilized at a low steady state. Overall acceptance closely tracked the constraint‐pass rate, indicating that structural and evolutionary plausibility filters, rather than stochastic rejection, dominated the search bottleneck. This behavior further suggests that the search was limited primarily by feasibility constraints, not by premature trapping within a purely greedy local optimum. Budget size determined the long‐term mobility of the optimization. After excluding a ten‐step burn‐in period, statistical analysis confirmed significant differences across budgets for both constraint‐pass rates (Kruskal–Wallis *H* = 17.06, *p* = 1.98 × 10^−4^) and overall proposal acceptance (*H* = 16.97, *p* = 2.06 × 10^−4^). Pairwise comparisons with Benjamini–Hochberg correction showed that the five‐substitution budget maintained significantly higher acceptance rates than the single‐substitution baseline (*q* = 1.48 × 10^−4^), while the three‐substitution budget also exhibited reduced mobility relative to the maximal allowance (*q* = 3.56 × 10^−2^). Consistent with this interpretation, increasing the mutation budget beyond three substitutions did not translate into better final designs because the enlarged search space remained sharply constrained by structural deviation and evolutionary plausibility requirements. These dynamics indicate that fold‐preserving suppressor mutations occupy a narrow region of sequence space that requires sufficient combinatorial flexibility to explore without violating structural constraints.

Given this constrained search landscape, we next examined whether independent optimization trajectories converged on shared structural vulnerabilities. Per‐residue mutation frequencies were quantified across top‐performing designs and compared with a budget‐matched random‐walk null model (Figure 8d). The evolutionary search displayed strong positional convergence, concentrating mutations at a limited set of structurally leveraged sites. Statistical enrichment analysis using Wilson CI and Fisher exact testing with Benjamini–Hochberg correction identified several residues under consistent selection pressure (adjusted *q* < 0.05). Residue 68 showed the strongest enrichment (OR 28.43, *q* = 0.026), while residues 80 and 95 formed an additional enriched cluster (OR 20.89, *q* = 0.049). These positions map to structured regions implicated in SOD1 stability and loop dynamics, suggesting that aggregation suppression arises from targeted perturbation of a limited set of structurally sensitive nodes. Among these enriched hotspots, residues 68, 80, and 95 were prioritized for experimental validation to determine whether the predicted reductions in aggregation propensity could be reproduced in cellular and biophysical assays (Figure S9).

To evaluate the robustness of these engineered suppressors against conformational fluctuations, we subjected 30 top‐performing designs and 30 matched random controls to structural perturbation tests incorporating coordinate jitter, feature noise, and edge dropout. Efficacy retention was defined as the fraction of perturbed states preserving a suppression margin of Δ*Risk* < −0.003 (Figure 8e). Designed variants maintained a median retention probability of 35%, whereas random controls showed a median of 0%. All 30 designed variants retained protective efficacy in at least one perturbed state, whereas 19 of the 30 random variants failed entirely under identical conditions. This difference produced a median retention shift of 0.35 (bootstrap 95% CI 0.258 to 0.667), with strong statistical separation (Mann–Whitney *U* = 770, *p* = 1.58 × 10^−6^) and a large Cliff's delta effect size of 0.71 (Figure 8e). These analyses reveal that aggregation suppression emerges from a sparse network of structurally tolerant residues, whereby a small number of coordinated substitutions can reduce aggregation propensity without disrupting the native fold.

### 2.11 Designed SOD1 Variants Suppress Aggregation While Retaining Native Stability

To translate these computational predictions into experimentally testable variants, candidate substitutions were ranked according to their mean predicted change in aggregation propensity across the design ensemble. The substitution V95N produced the largest predicted reduction (mean Δ*Risk* = −0.0118), followed by L68S (−0.0086) and R80L (−0.0076) (Supplemental data 1). Selecting the strongest suppressive substitution at each hotspot position yielded three prioritized suppressor candidates predicted to substantially reduce aggregation without compromising structural stability.

To experimentally evaluate these predictions, the corresponding substitutions were introduced into the human SOD1 coding sequence by site‐directed mutagenesis to generate recombinant constructs encoding the V95N, L68S, and R80L variants. Intracellular aggregation was assessed using a filter‐retardation assay that captures detergent‐insoluble SOD1 species retained on cellulose acetate membranes (Figure S9a). The ALS‐associated G93A mutant produced robust aggregate accumulation that exceeded WT levels by approximately fourfold (Figure S9b). In contrast, each designed variant substantially reduced aggregate retention relative to G93A. R80L showed an intermediate reduction, whereas L68S and V95N decreased aggregate capture to approximately one‐half and one‐quarter of the G93A signal, respectively, approaching WT levels.

To determine whether aggregation suppression occurred without destabilizing the native fold, thermal stability was measured using a SYPRO Orange thermal shift assay. The G93A mutant exhibited pronounced destabilization, with a melting temperature of 62.75°C ± 0.50°C compared with 72°C ± 1.08°C for WT SOD1 (*p* = 6.84 × 10^−5^) (Figure S9c,d). In contrast, the designed variants remained within the thermodynamic range of the native protein. R80L unfolded at 70.63°C ± 0.63°C and did not differ significantly from WT (*p* = 0.081). L68S showed a melting temperature of 73.25°C ± 1.32°C (*p* = 0.196), while V95N exhibited the highest stability at 73.75°C ± 0.50°C and a modest but significant increase relative to WT (*p* = 3.96 × 10^−2^) (Figure S9c,d). One‐way ANOVA confirmed an overall difference in melting temperature across variants (*p* = 8.57 × 10^−11^) (Figure S9d). These measurements indicate that sparse mutations identified by the generative design framework suppress intracellular aggregation while preserving the global thermodynamic stability of SOD1.

## 3 Discussion

Protein aggregation emerges from a dynamic interplay between local energetic frustration, residue‐level exposure, and long‐range structural coupling [26, 27]. Current computational predictors frequently collapse these dimensions into static descriptors or sequence‐derived heuristics [28, 29]. Earlier machine learning approaches, including our previous SKALE 1.0 framework, established that integrating physicochemical features improves performance over basic sequence‐only rules. However, these models conceptually treat proteins as collections of local attributes, and therefore remain limited in their ability to explain how distal substitutions reshape aggregation behavior without overtly disrupting the folded monomer [23]. This mechanistic gap is especially important in neurodegenerative proteins, where pathogenicity often reflects a subtle redistribution of aggregation competence rather than gross unfolding [30, 31, 32].

SKALE 2.0 addresses this limitation by recasting aggregation propensity as a phase‐resolved geometric problem. Instead of reducing mutational effects to a binary risk axis, the framework learns how substitutions perturb the structural manifold connecting WT and mutant states across distinct kinetic regimes. By representing each protein as a multimodal graph that couples three‐dimensional coordinates to solvent accessibility, hydrogen‐bond organization, normal mode stiffness, and evolutionary embeddings, and by processing these features through a Siamese EGNN with phase‐gating tokens, SKALE 2.0 explicitly separates nucleation from elongation while preserving sensitivity to mutation‐induced geometric distortions. The resulting latent representation is not merely a compressed feature space but a biophysical coordinate system in which structural deviation, kinetic phase, and aggregation risk become jointly interpretable.

Several findings from this framework refine how aggregation should be conceptualized. First, the latent manifold is organized primarily by protein family, indicating that SKALE 2.0 learns global fold constraints before resolving mutation‐specific perturbations. Within this family‐aware structure, mutation trajectories remain directional and quantifiable through latent displacement, enabling aggregation‐linked structural divergence to be measured continuously rather than binarized. Second, phase‐token projections revealed a conserved directional shift from nucleation to elongation across the evaluated proteins, suggesting that progression through aggregation is encoded as a shared geometric transformation despite major differences in fold architecture. Third, elongation is consistently more sensitive than nucleation to pathogenic perturbation. This observation supports the view that many disease‐associated mutations do not simply trigger aggregation initiation but instead disproportionately amplify the structural conditions that stabilize and propagate growing assemblies once nucleation has occurred [33, 34].

Phase‐resolved saliency analyses further showed that nucleation and elongation obey different structural rules. Nucleation preferentially emphasizes buried hydrophobic regions, whereas elongation preferentially emphasizes solvent‐accessible surfaces. This redistribution is consistent with a temporally ordered mechanism where early aggregation depends on destabilizing or exposing cryptic internal vulnerabilities, while later growth increasingly depends on externally accessible interfaces competent for templating and fibril recruitment [35, 36, 37]. Importantly, this phase partitioning is not restricted to a single scaffold. Across the validation set, SKALE 2.0 recovered both phase‐aligned and phase‐decoupled archetypes, indicating that aggregation phenotypes cannot always be reduced to a single monotonic risk axis. Some variants perturb both kinetic stages in the same direction, whereas others create a genuine mismatch between the initiation barrier and the rate of subsequent fibril growth.

This distinction proved experimentally meaningful. In recombinant SOD1, A96Y behaved as a phase‐concordant suppressor that delayed aggregation onset and reduced propagation, whereas I105N acted as a phase‐concordant enhancer that accelerated both processes beyond the G93A disease control. Most notably, K4N reproduced the predicted phase‐switch phenotype by strongly delaying lag time while simultaneously increasing the propagation rate and final amyloid burden once assembly had initiated. These data demonstrate that phase decoupling is not merely a latent space abstraction. It corresponds to measurable kinetic behavior in vitro and provides a mechanistic vocabulary for distinguishing variants that reduce nucleation from those that reduce overall aggregate load, outcomes that are often conflated in conventional prediction schemes [19, 38].

A further advance of SKALE 2.0 is its ability to expose cryptic aggregation liability not captured by structural confidence or global destabilization metrics. Residue‐level analysis of SOD1 G86R showed that high aggregation sensitivity can persist even in regions with high AlphaFold confidence, arguing against a simple equivalence between low confidence, disorder, and aggregation risk [39, 40]. Pathogenic substitutions redistributed sensitivity across distal surface patches rather than confining effects to the mutation site itself. At the whole‐protein level, variants with strong predicted aggregation risk frequently exhibited only modest global structural ripple strength, indicating that high‐risk states can emerge from localized rewiring of exposed interfaces without widespread structural collapse. This distinction helps explain why some neurodegenerative mutants remain folded and stable while nevertheless acquiring potent assembly competence [30, 31, 34].

The saturation mutagenesis atlases and motif‐level analyses extend this view by showing that aggregation landscapes are structured at multiple resolutions. Across SOD1, TDP‐43, MAPT, and PRNP, part of the substitution effect reflected broad amino acid‐specific biases, yet scaffold‐specific hotspots remained after normalization, revealing localized zones of true structural sensitivity. In SOD1, these hotspots concentrated within functionally meaningful regions such as the zinc‐binding loop, the electrostatic loop, and the amyloidogenic core, where the sign and magnitude of mutational effects depended strongly on kinetic phase and local context. Aggregation vulnerability is therefore neither uniformly distributed across sequence nor reducible to canonical amyloid motifs alone [28, 30, 41]. It is embedded within a residue grammar shaped by fold topology, solvent exposure, and phase‐specific coupling to the learned aggregation manifold.

These same properties make the framework useful not only for interpretation but also for design. Because the latent space learned by SKALE 2.0 is differentiable and structurally constrained, it can be inverted to search for suppressor mutations that reduce aggregation while preserving fold integrity and sequence plausibility. At the single‐site level, gradient‐guided perturbation identified distal allosteric positions such as residue 58 in SOD1, where substitutions shifted latent trajectories toward the WT basin. At the combinatorial level, evolutionary optimization revealed that the deepest safe suppressors were not distributed across broad mutational space but concentrated within a narrow, structurally tolerant regime. The emergence of a strong Pareto frontier, the superiority of a three‐substitution budget over both one‐substitution and five‐substitution searches, and the convergence on residues 68, 80, and 95 together indicate that robust suppression is governed by a sparse network of structurally leveraged nodes rather than diffuse sequence redesign. Experimental validation of V95N, L68S, and R80L confirmed this principle, showing reduced intracellular aggregation together with preservation and, in one case, modest improvement of native thermal stability.

These observations carry broader implications for therapeutic design. First, they suggest that protective interventions need not rely on wholesale stabilization of the native fold. Selectively dampening a small number of aggregation‐permissive interfaces may be sufficient to redirect the protein away from high‐risk trajectories. Second, they imply that aggregation suppression is constrained but not fragile. Designed suppressors retained efficacy under structural perturbation far more effectively than random controls, indicating that the model is not merely exploiting isolated local minima in sequence space. Third, the shared organization of phase trajectories across distinct amyloidogenic proteins raises the possibility that general geometric principles underlie pathogenic self‐assembly even when the molecular details of each scaffold differ substantially [34, 37, 42].

Several limitations remain. The present implementation is anchored to static AlphaFold‐derived structures and therefore cannot fully represent conformational heterogeneity, rare excited states, post‐translational modifications, or environmental variables that modulate aggregation in cells [39, 43, 44]. Because the framework extracts geometry directly from these inputs, predictive performance depends on the coordinate fidelity of the provided structural model. Furthermore, physicochemical conditions such as pH [45] and ionic strength [46] are not explicitly parameterized, meaning SKALE 2.0 operates within condition‐bounded structural contexts rather than functioning as a universal predictor across arbitrary solution environments. The latent phase labels are also learned from kinetic supervision rather than directly observed structural intermediates, so their mechanistic interpretation remains an inferred mapping onto the aggregation process, despite being strongly supported by experiment. Although the computational framework was evaluated across multiple aggregation‐prone proteins, including SOD1, TDP‐43, MAPT, and PRNP, the current experimental validation remains concentrated in the SOD1 system. In this setting, the recombinant kinetic measurements and follow‐up suppressor experiments provide direct support for the predicted phase‐concordant suppressor, phase‐concordant enhancer, and phase‐switch behaviors. However, equivalent biochemical validation has not yet been established for TDP‐43, MAPT, or PRNP at the same depth. The broader applicability of SKALE 2.0 across these scaffolds should therefore be interpreted as computationally supported, pending matched experimental validation. A related boundary concerns the biological class of aggregation being modeled. The present training context, experimental validation, and suppressor‐design logic are anchored exclusively to pathological aggregation phenotypes. While functional amyloids share ordered assembly features, they operate under distinct regulatory constraints and physiological binding partners [47, 48, 49]. The applicability of SKALE 2.0 to functional assemblies therefore requires dedicated benchmarking and task‐specific retraining before broader generalization can be assumed. Future integration of ensemble‐based structural sampling, cryo‐EM‐derived intermediate structures, and richer biophysical readouts should sharpen both residue‐level attribution and phase assignment. It will also be important to extend validation beyond SOD1 through broader mutational scans and, where feasible, cellular and in vivo models to determine how broadly this sparse design logic generalizes across distinct structural classes.

Within these bounds, SKALE 2.0 establishes a framework in which aggregation is no longer treated as a single end‐state property but as a geometry of phase‐dependent structural susceptibility that can be measured, interpreted, and engineered. By linking latent structural distortion to experimentally observable changes in lag time, propagation, aggregate burden, and fold stability, the model bridges atomic topology with disease‐relevant kinetic behavior. More broadly, it provides a route from mechanistic prediction to constraint‐aware suppressor design, offering a foundation for programmable intervention in protein aggregation disorders.

## 4 Methods

### 4.1 Plasmid Construction and Recombinant Protein Expression

Plasmids encoding the SOD1 structural variants WT, K4N, L68S, R80L, G93A, V95N, A96Y, and I105N were designed in‐house and cloned into pET‐28a or pcDNA3.1(+)‐Flag expression vectors using standard molecular biology methods. The pcDNA3.1(+)‐Flag SOD1 WT and G93A constructs were generated previously [50, 51]. Recombinant protein expression and purification were performed as previously described [52], with targeted modifications to improve protein yield and promote proper metallation. Chemically competent *Escherichia coli* BL21(DE3) cells (NEB) were co‐transformed by heat shock with plasmids encoding SOD1 together with the yeast copper chaperone CCS [53], and transformants were selected using appropriate antibiotics. Single colonies were expanded in starter cultures and scaled into bulk expression cultures. Protein expression was induced at mid‐log phase with IPTG, and the induction medium was supplemented with copper and zinc to promote co‐translational metallation and stabilization of the native fold. Bacterial cells were collected by centrifugation and lysed by sonication or high‐pressure homogenization in a buffered aqueous solution containing protease inhibitors. Following lysate clarification by centrifugation, soluble SOD1 was enriched by ammonium sulfate fractionation. The SOD1‐containing fraction was then purified by sequential size‐exclusion chromatography on a HiLoad 16/60 Superdex 75 pg column (Cytiva) and anion‐exchange chromatography on a HiTrap DEAE column (Cytiva). A standard salt gradient was applied during the anion‐exchange step to separate SOD1 from residual contaminants. Protein purity was evaluated by SDS‐PAGE, and protein concentration was determined using a bicinchoninic acid assay [54].

### 4.2 Thioflavin Fluorescence Aggregation Assay

Aggregation kinetics of five recombinant SOD1 variants (WT, G93A, A96Y, K4N, and I105N) were monitored using a ThT fluorescence assay in a high‐throughput microplate format. Reaction mixtures contained 30 µm SOD1 dimer in 1 × PBS at pH 7.4. To promote disulfide reduction and complete metal chelation before fibrillization, the buffer was supplemented with 20 mm dithiothreitol (DTT), 5 mm EDTA, and 10 µm ThT. Samples were dispensed into clear‐bottom 384‐well microplates at a final volume of 50 µL per well. The plates were incubated at 37°C for 30 min to allow structural reduction and were then sealed with optically clear adhesive film to prevent evaporation during the extended kinetic assay. Fibrillization was induced under controlled agitation within a microplate reader maintained at 37°C. Fluorescence acquisition was performed every 900 s, with each cycle preceded by 330 s of double‐orbital shaking at 300 rpm. ThT fluorescence was quantified using excitation and emission wavelengths of 440 and 490 nm, respectively, to capture the full temporal trajectory of amyloid assembly.

### 4.3 Filter Retardation Assay

Filter retardation assays were performed as previously described [23]. In brief, transfected HEK293T cells were lysed under denaturing conditions, heated to dissociate soluble species, and clarified by centrifugation. Equal amounts of protein were filtered through 0.2 µm cellulose acetate membranes using a vacuum apparatus to retain SDS‐insoluble aggregates. Membranes were then washed, blocked, and immunodetected with anti‐FLAG primary antibody and HRP‐conjugated secondary antibody. Aggregate‐associated signal was quantified after background correction, and data are shown as mean ± s.e.m. (standard error of the mean) from at least three independent biological replicates.

### 4.4 Differential Scanning Fluorimetry Assay

Thermal stability of recombinant SOD1 variants was measured using a fluorescence‐based thermal shift assay with SYPRO Orange dye (ThermoFisher) [55, 56]. Purified SOD1 proteins were diluted in PBS at pH 7.4 to a final concentration of 5 µm in a total reaction volume of 20 µL. SYPRO Orange was added to a final concentration of 5× relative to the manufacturer's stock solution. Protein and dye solutions were prepared immediately before measurement to minimize background fluorescence and ensure consistent dye binding. Thermal unfolding was monitored using the BioRad CFX96 real‐time PCR instrument. Fluorescence signals were recorded continuously while the temperature was increased from 25°C to 95°C at a ramp rate of 0.5°C per minute. Excitation and emission settings were configured according to the instrument parameters optimized for SYPRO Orange detection. Thermal unfolding curves were obtained by plotting fluorescence intensity as a function of temperature. The apparent melting temperature (*T_m_*) for each variant was calculated from the midpoint of the unfolding transition, determined by fitting the derivative of the fluorescence curve. Each measurement was performed with three independent replicates prepared from separately purified protein samples. Statistical comparisons of melting temperatures across variants were performed using one‐way analysis of variance, followed by pairwise comparisons where appropriate. All reported values represent the mean ± s.e.m calculated from independent replicates.

### 4.5 Geometric Graph Representation and Feature Embedding

Protein structures were formalized as geometric graphs *G* = (*V, E*), where nodes *V* represent Cα atoms and edges *E* define spatial connectivity. Graph connectivity was constructed dynamically by connecting all node pairs (*i*, *j*) satisfying a Euclidean distance threshold ∣∣*x_i_* − *x_j_* ∣∣_2_ < 10 Å, subject to a maximum degree constraint (*k *= 32) to enforce local sparsity [57].

Each node *i* was initialized with a high‐dimensional feature vector h*_i_* ^(0)^ ∈ $R^{d_{in}}$ composed of three primary feature classes. First, geometric descriptors, including hydrogen‐bond distances and SASA, were computed as previously described [23] and encoded as residue‐level geometric attributes. Second, conformational flexibility was quantified from molecular dynamics simulations as the Cα root mean square fluctuation (RMSF). Structures were solvated in explicit solvent and simulated in OpenMM using the AMBER14 force field under periodic boundary conditions, using particle mesh Ewald electrostatics [58, 59], hydrogen‐bond constraints, and Langevin dynamics [60] at 300 K. Following minimization, equilibration, and production sampling, protein Cα trajectories were aligned to the reference structure and RMSF values were calculated with MDTraj [61]. These RMSF values were assigned to the corresponding graph nodes as dynamic flexibility descriptors. Third, protein language model features were incorporated by extracting frozen per‐residue embeddings using ESM2 (esm2_t6_8M_UR50D) [62, 63]. Full protein sequences were tokenized, final‐layer representations were extracted, and residue‐level embeddings were obtained by aligning the token sequence to the protein sequence after excluding special tokens. Embeddings were cached to disk to avoid recomputation and then concatenated with the structural and physicochemical descriptors to form the final node input matrix.

### 4.6 Dataset Composition, Supervision Coverage and Split Strategy

The structural cohort comprised four protein families associated with neurodegenerative aggregation, namely SOD1, TDP‐43, MAPT and PRNP. The training manifest contained nine structure entries, including four WT reference structures and five matched mutant structures corresponding to SOD1 G86R, TDP‐43 S332N, MAPT P301L, and PRNP E200K and G127V. These entries were organized into five WT‐to‐mutant pairs for Siamese encoding. Cohort composition, entry‐level supervision status, and pair structure are summarized in Table S1. The aggregation‐risk branch was implemented as a conditional head that was activated only when binary aggregation labels were provided in the manifest. In the present training configuration, binary risk labels were not supplied, and this branch was therefore not directly supervised. Model evaluation used a fixed random holdout applied at the pair level, with 75% of pairs assigned to training and 25% assigned to validation using a random seed of 42. Target standardization was fitted on the training subset and then applied to the validation subset.

### 4.7 Siamese EGNN

To distinguish mutation‐induced structural strain from rigid‐body variations, we implemented a paired WT‐to‐mutant Siamese architecture with shared weights. For each protein pair, the WT and matched mutant structure were processed through the same EGNN message‐passing backbone, allowing both structures to be represented in a common latent space while preserving sensitivity to mutation‐induced geometric perturbations. At each layer *l*, node embeddings were updated using both residue‐level features and geometric edge distances, with message passing and coordinate updates defined as follows.

Equivariant edge messages m*_ij_* were computed from the node features of neighboring residues and their rotation‐invariant squared distance:

(1) $$m_{ij}=\phi_{e}\left(h_{i}^{\left(l\right)},{\;h}_{j}^{\left(l\right)},\;∥x_{i}^{\left(l\right)}-x_{j}^{\left(l\right)}{∥}^{2}\right)$$

where *ϕ_e_* is a multilayer perceptron. To model structural relaxation and mutation‐induced strain, atomic coordinates x*_i_* were updated through a radial vector field derived from the learned messages:

(2) $$\begin{array}{c} x_{i}^{\left(l+1\right)}=x_{i}^{\left(l\right)}+\sum_{j\in N(i)}\left(x_{i}^{\left(l\right)}-x_{j}^{\left(l\right)}\right)\times {⌀}_{x}\left(m_{\mathrm{ij}}\right) \end{array}$$

where N(i) denotes the neighbor set of node *i*. This update rule preserves *E*(3) equivariance, such that coordinate outputs transform consistently under global rotations and translations of the input structure. Node features were subsequently updated by aggregating incoming messages from neighboring residues:

(3) $$h_{i}^{\left(l+1\right)}=\emptyset_{h}\;\left(h_{i}^{\left(l\right)},\sum_{j\in N\left(i\right)}m_{\mathrm{ij}}\right)$$

where *ϕ_x_* and *ϕ_h_* are distinct non‐linear mapping functions. This weight‐shared Siamese design ensures that WT and mutant structures were evaluated under an identical geometric transformation, enabling mutation‐induced latent shifts to reflect structural perturbation rather than differences in model parameterization.

### 4.8 Phase‐Gating via FiLM

To represent phase dependence, we introduced a discrete phase token that distinguishes nucleation and elongation and used FiLM to condition the learned structural representations. The phase identifier was embedded as a learnable vector, *e_phase_*, and passed through two projection functions to generate affine modulation parameters γ and β, which transformed the latent node embeddings after the final EGNN update:

(4) $$\gamma =F_{\gamma }\left(e_{phase}\right),\;\beta =F_{\beta }\left(e_{phase}\right)$$

(5) $$h_{\mathrm{gated}}=\left(1+\gamma \right)⊙\;h_{\mathrm{late}\mathrm{nt}}+\beta$$

where ⊙ denotes the Hadamard product. This conditioning scheme allowed the same geometry‐aware backbone to generate phase‐specific representations for distinct aggregation stages while retaining a shared structural latent space. Phase‐conditioned node embeddings were then summarized into graph‐level latent vectors using masked pooling, yielding *Z_WT_* (*phase*) and *Z_mut_* (*phase*) for each WT and mutant pair. The phase‐conditioned embedding shift was defined as:

(6) $$\Delta Z\left(phase\right)=Z_{mut}\left(phase\right)-Z_{WT}\left(phase\right)\;$$

This latent shift served as the core quantity for phase‐resolved mechanistic analysis, allowing mutation‐induced structural displacement to be compared separately across nucleation and elongation regimes.

### 4.9 Output Heads and Multi‐Objective Loss Function

Three prediction heads were attached to the phase‐conditioned latent representation to guide optimization. The kinetics head was implemented as a three‐output regressor that predicted lag time, growth rate, and plateau intensity directly from the phase‐conditioned latent state *z*. The auxiliary reconstruction head served as a node‐level decoder that projected latent node embeddings back into the original input feature space, reducing information loss and stabilizing representation learning. The aggregation risk head was implemented as a global binary classifier that predicted aggregation probability and was activated only when binary aggregation labels were available in the training manifest.

The model was optimized using a composite objective function designed to align latent geometry with experimentally measured kinetic rates. The total loss $L_{total}$ was formulated as:

(7) $$L_{total}=L_{kin}+\lambda_{aux}L_{aux}+\lambda_{risk}\;L_{risk}$$

where $L_{kin}$ employed an MSE term to minimize the divergence between predicted kinetic parameters ($\hat{y}$) and experimentally measured targets (*y*). This head was trained on samples with available kinetic labels, with target parameters comprising lag time *τ*, growth rate *k*, plateau intensity *A*. These targets were z‐score standardized using parameters fitted on the training split and then applied to the validation split before model training. The auxiliary reconstruction loss ($L_{aux}$), weighted by λ_*aux*_  =  0.2, encouraged recovery of the original physicochemical input features from the latent representation and was included to reduce feature collapse. The conditional aggregation risk loss ($L_{risk}$) utilized a binary cross‐entropy term applied to the global risk logit. This term was enabled when binary aggregation labels were provided (λ_*risk*_  = 1) and disabled otherwise (λ_*risk*_ = 0), allowing the framework to support self‐supervised geometric learning when risk labels were unavailable.

### 4.10 Representative Baseline Benchmarking and Phase‐Resolution Evaluation

The trained framework was benchmarked against three simplified baselines evaluated on the same cohort. The protein language model baseline used mean‐pooled ESM2 embeddings alone. The AlphaFold‐derived feature baseline used mean‐pooled solvent accessibility, hydrogen‐bond, and conformational fluctuation features. The structure‐based deep learning baseline retained the shared EGNN backbone but omitted phase gating, using the pooled latent representation before phase‐conditioned modulation. SKALE 2.0 was evaluated using phase‐conditioned graph‐level latent representations under nucleation and elongation tokens. For each WT‐to‐mutant pair, phase‐dependent mutation modulation was quantified from the phase‐specific latent displacements, with $d_{P_{0}}=∥Z_{mut}^{P_{0}}\;-\;Z_{WT}^{P_{0}}∥$ and $d_{P_{1}}=∥Z_{mut}^{P_{1}}\;-\;Z_{WT}^{P_{1}}∥$. The phase modulation index was defined as $|d_{P_{1}}-\;d_{P_{0}}|$. Mean mutation impact in nucleation and elongation was reported as the cohort mean of $d_{P_{0}}$ and $d_{P_{1}}$, respectively, with s.e.m. calculated across WT‐to‐mutant pairs. Phase separability was evaluated using a linear phase‐probe classifier on standardized nucleation and elongation embeddings, together with the silhouette score from the same latent representations. For non‐phase‐aware baselines, identical representations were supplied to both phase labels.

### 4.11 Latent Manifold Analysis and Inverse Design

The magnitude of mutation‐induced structural perturbation was quantified as the Euclidean distance between WT and mutant embeddings in the StandardScaler‐normalized, phase‐conditioned latent manifold:

(8) $$∥\Delta Z∥=∥\mathrm{Pool}\left(h_{mut}\right)\;-\;\mathrm{Pool}\left(h_{WT}\right){∥}_{2}$$

where Pool denotes graph‐level pooling of node embeddings. For rational design, we used gradient‐guided in silico mutagenesis to identify substitutions predicted to reduce aggregation risk while preserving latent structural fidelity. Sensitivity was computed from gradients of the predicted aggregation‐risk logit with respect to the input features (∇_*x*_Logit) rather than the probability score, thereby reducing the effect of vanishing gradients in saturated regimes. To estimate the relative contribution of each biophysical modality, feature sensitivities were normalized to the mean sensitivity of the conformational dynamics channel. Gradients were computed under the elongation‐phase token to capture aggregation‐prone dynamics associated with fibril growth. Raw residue‐level saliency profiles were used without spatial smoothing to preserve residue‐level structural resolution. Risk shifts were reported in normalized aggregation sensitivity units. Candidate suppressor mutations were identified by iteratively perturbing sequence in the direction of −∇_x_ *R*, corresponding to gradient descent on the predicted aggregation‐risk objective, to prioritize substitutions that minimize aggregation risk while maintaining latent structural fidelity.

### 4.12 Structural Risk Mapping and Biological Validation

To map latent aggregation risks onto physical structures, we computed residue‐level risk sensitivity scores as the L2 norm of the gradient of the global risk logit with respect to the per‐residue node feature vector, defined as ∥∂Logit/∂x∥_2_. Gradients were computed under the elongation phase token to capture aggregation‐prone dynamics associated with fibril growth. For stability‐risk landscape analysis, sensitivity scores were min–max normalized to the range 0–1. Local structural confidence, measured as predicted local distance difference test (pLDDT), was extracted from the B‐factor field of the AlphaFold‐predicted structure file in Protein Data Bank (PDB) format by averaging B‐factor values across all atoms within each residue. For mutation‐induced risk redistribution analysis, WT and mutant sensitivity profiles were normalized by their respective maximum values to place both profiles on comparable dynamic ranges before computing the differential risk‐shift profile. Where necessary, profiles were truncated to the minimum shared sequence length to ensure residue‐wise alignment.

To visualize risk on the three‐dimensional structure, residue‐level sensitivity scores were rescaled to the range 0–100 and written into the B‐factor column of the target PDB‐format structure file. The structure was then rendered in PyMOL, with the surface colored according to the B‐factor field to highlight regions of elevated aggregation sensitivity. Structural Ripple Strength was quantified as the L1 distance between the max‐normalized WT and mutant risk sensitivity profiles, as follows:

(9) $$\begin{array}{ccc} & & \text{Ripple Strength}=\sum_{i=1}^{n}\left|\frac{S_{\mathrm{mut}}^{\left(i\right)}}{\max\left(S_{\mathrm{mut}}\right)+\varepsilon }-\frac{S_{\mathrm{wt}}^{\left(i\right)}}{\max\left(S_{\mathrm{wt}}\right)+\varepsilon }\right|,\\ & & \varepsilon =10^{-6} \end{array}$$

where *S* ^(*i*)^ is the residue‐wise risk sensitivity (∥∂Logit/∂*f_i_* ∥_2_), and profiles were truncated to the minimum common length n for alignment. This metric was correlated against the global risk logit predicted by the model's elongation‐phase (*P*_1_) risk head for each mutant.

### 4.13 Statistical Analysis

Statistical comparisons of phase‐specific attention weights were performed using two‐sided Welch's *t*‐tests. These tests treat residues as independent observations and were reported to illustrate effect size rather than support population‐level inference. Kinetics supervision was restricted to the available WT kinetic subset, and predictive accuracy for kinetic trajectories was assessed using the MSE of parameter‐level predictions on the standardized scale rather than full ThT curve reconstruction. Uncertainty in latent space stability (Figure 2a) was quantified via bootstrapping with noise injection over 30 iterations, whereas uncertainty in biophysical feature sensitivity (Figure 2d) was estimated as the s.e.m. across the validation cohort. All optimization was performed using the AdamW algorithm with a learning rate *η* = 2 × 10^−4^, and weight decay *λ *= 1 × 10^−3^ on NVIDIA GPUs.

### 4.14 In Silico Saturation Mutagenesis and Structural Encoding

To map the mutational landscapes of the target proteins, we generated an in silico saturation mutagenesis atlases by systematically evaluating all possible single‐amino acid substitutions with the trained SKALE 2.0 model under a specific phase‐conditioning regime. To enable rapid exhaustive screening, substitutions were introduced directly into the localized node feature representations. For nodes containing explicit one‐hot amino acid identity encodings, the WT identity channel was reassigned to the target substitution. Where explicit identity channels were not available, substitutions were approximated using a deterministic proxy transition based on fixed orthogonal amino acid basis vectors, thereby preserving relative identity shifts in feature space. The modified node features, together with the WT spatial coordinates and graph topology, were then processed through the EGNN backbone. The resulting embeddings were gated by the targeted aggregation phase token and mapped to a global risk logit. The specific substitution effect was quantified as the difference in the predicted risk logit between the mutated and WT states:

(10) $$\Delta {\mathrm{Risk}}_{i,a}={\mathrm{logit}}_{\mathrm{mut}\left(i,a\right)}-{\mathrm{logit}}_{\mathrm{WT}\left(i,a\right)}$$

To stabilize visual contrast across diverse protein targets, extreme outlier values within the resulting substitution matrices were bounded to upper and lower empirical quantiles.

### 4.15 Stochastic Perturbation and Uncertainty Quantification

To ensure the identified mutational effects were robust against structural uncertainty, the entire atlas was recomputed across multiple perturbation replicates. These stochastic evaluations incorporated isotropic Gaussian noise applied to both the node features (*σ*_feat_) and the spatial coordinates (*σ*_coord_), alongside probabilistic edge dropout to simulate dynamic variations in graph connectivity. The final risk shift for each substitution was defined as the median value across all replicates, while predictive uncertainty was formally quantified using the median absolute deviation. Substitutions falling within the highest decile of uncertainty were explicitly marked to prevent the overinterpretation of highly variable predictions.

### 4.16 Positional Mutation Tolerance and Directional Bias

Residue‐specific mutational tolerance was calculated to capture the typical magnitude of aggregation‐risk sensitivity at each sequence position. This index was defined as the trimmed mean of absolute risk shifts across the 19 possible non‐native substitutions, excluding the most extreme upper decile to reduce the influence of individual hypersensitive substitutions

(11) $$\mathrm{Toleranc}e_{i}=\frac{1}{\left|A_{\mathrm{trimmed}}\right|}\;\sum_{a\in A_{\mathrm{trimmed}}}\left|\Delta \mathrm{Ris}k_{i,a}\right|$$

where A_trimmed_ denotes the retained substitution set after upper‐decile trimming. CI (95%) was estimated by bootstrap resampling across perturbation replicates. Smoothed positional profiles were generated using a centered rolling mean. Directional bias at each residue was quantified as the net difference between the fraction of substitutions that increased aggregation risk and the fraction that reduced aggregation risk, thereby distinguishing positions biased toward risk amplification from those biased toward suppression.

### 4.17 Global Stability and Functional Hierarchy Validation

The global reliability of each mutational atlas was evaluated using effect‐level, rank‐level, and sign‐level stability metrics. Effect‐level consistency was quantified by Spearman correlation between the unperturbed reference atlas and each structurally perturbed replicate. To assess whether the functional hierarchy of aggregation hotspots was preserved under perturbation, rank‐level stability was evaluated by isolating the top fraction of risk‐driving residues and computing the Jaccard similarity index across perturbation iterations:

(12) $$J\left(S_{clean},\;S_{perturbed}\right)=\frac{\left|S_{clean}\cap S_{perturbed}\right|}{\left|S_{clean}\cup S_{perturbed}\right|}$$

Where *S_clean_* denotes the hotspot set from the unperturbed reference atlas and *S_perturbed_* denotes the corresponding hotspot set from each perturbed replicate. The statistical significance of hotspot retention was assessed by permutation testing, in which the observed median Jaccard overlap was compared with a null distribution generated from randomly sampled residue subsets of identical cardinality. Sign‐level stability was quantified as the fraction of perturbed predictions that retained the same risk directionality as the unperturbed reference atlas.

### 4.18 Phase‐Dependent Aggregation Switch Motifs

Phase‐dependent aggregation switch motifs were mapped using single‐residue substitution scans under explicit phase conditioning. For high‐fidelity analysis of WT SOD1, the structural feature block was held fixed while protein language model embeddings were independently regenerated for each mutated sequence using the ESM2 architecture. Substitution effects were quantified as predicted aggregation‐risk shifts relative to the WT baseline under both nucleation and elongation phase tokens. A phase‐switcher statistic was defined to identify residues exhibiting a sign inversion in predicted risk between phases, with the switch magnitude quantified as the minimum absolute effect size across the two kinetic states. In parallel, absolute phase divergence was measured as the magnitude of the difference between phase‐specific effects ($|\Delta \mathrm{Ris}k_{P_{1}}\;-\;\Delta \mathrm{Ris}k_{P_{0}}|$). Statistical significance was assessed using a paired permutation procedure in which phase labels were shuffled to construct empirical null distributions, followed by Benjamini–Hochberg FDR correction across all residues. Final mutational calls required robust retention under bootstrap resampling of amino acid choices, with a minimum stability frequency of 80% used to filter isolated computational artefacts.

### 4.19 Motif Window Construction and Positional Preference Landscapes

Motif windows were constructed to contextualize phase‐dependent mutational switches at the local sequence level. Contiguous structural windows were centered on high‐scoring seed residues, with spacing constraints applied to prevent artefactual merging of distinct sequence–interaction interfaces. Within each selected window, raw mutational shifts were converted into probability distributions over the amino acid vocabulary using a temperature‐scaled softmax function. The temperature parameter *τ* was defined dynamically from the median absolute effect magnitude within each window to improve interpretability and stabilize variance across heterogeneous positions. The preference probability *p* for amino acid *a* at position *i* was calculated by:

(13) $$p_{i,a}=\frac{\exp\left(\Delta \mathrm{Ris}k_{i,a}/\tau \right)}{\sum_{j}\exp\left(\Delta \mathrm{Ris}k_{i,j}/\tau \right)}$$

The stringency of amino acid preference at each sequence position was quantified using IC, defined as:

(14) $${\mathrm{IC}}_{i}=\log_{2}\left(20\right)+\sum_{a}p_{i,a}\log_{2}\left(p_{i,a}\right)$$

where 20 denotes the amino acid alphabet size. Positional phase divergence was quantified using JS divergence between the nucleation‐ and elongation‐conditioned probability distributions, with statistical support estimated from permutation‐derived probability values. Regional shifts in preference strength between phases were assessed across each contiguous window using paired Wilcoxon signed‐rank tests.

### 4.20 Architecture‐Dependent Structural Context and Global Geometric Consensus

The structural context of phase‐dependent mutational sensitivity was investigated by integrating residue‐level sensitivity profiles across the evaluated protein cohort. To enable rapid cross‐protein screening, deterministic amino acid perturbation basis vectors were used to generate proxy substitution matrices for targets lacking high‐fidelity language model rescoring. Within each protein, highly divergent hotspots were defined from the top decile of phase divergence, ensuring comparable site selection independent of raw predictive scale. Solvent accessibility was computed directly from the reference atomic coordinates using the Shrake–Rupley algorithm and binarized using a within‐protein median split. Proteins were then stratified into structured and disordered architectural classes to test whether mutational hotspots preferentially occupied solvent‐exposed environments. Enrichment was quantified using the log odds ratio and assessed for significance using Fisher's exact tests. To determine whether geometric biases persisted beyond architecture‐specific stratification, cohort‐wide geometric consensus was visualized by comparing the solvent‐accessibility distributions of identified hotspots with the non‐sensitive background population using kernel density estimation.

### 4.21 Constraint‐Aware Computational Evolution

Constraint‐aware suppressor discovery was performed by exploring sequence space under explicit phase conditioning while preserving a fixed structural architecture. For each proposed variant, the localized node input matrix was constructed by concatenating the fixed structural feature channels with sequence‐specific embeddings generated by the ESM2 protein language model, while synchronizing explicit amino acid one‐hot encodings to the proposed sequence. Sequence exploration was implemented as a budgeted Markov Chain Monte Carlo sampling procedure in which the mutation budget defined the maximum permitted Hamming distance from the starting sequence and therefore the accessible search space around the seed variant. Each proposal was evaluated across three coupled quantities. Aggregation suppression was defined as the phase‐conditioned risk logit shift Δ*Risk* = *logit*_mut _ − *logit*_WT_. Structural cost was quantified as the Euclidean distance between pooled latent vectors, ‖Δ*z*‖  =  ‖*z*_mut_  −  *z*_WT_‖_2_. Evolutionary plausibility was calculated as the summed log‐probability shifts across mutated positions using a WT conditional pseudo‐log‐likelihood score, Δ*PLL*. Structural cost and evolutionary plausibility were applied first as hard feasibility filters, such that only proposals satisfying the predefined maximum structural deviation and minimum plausibility thresholds were eligible for acceptance. Within this feasible set, accepted moves were determined by a Metropolis criterion driven by aggregation risk, with uphill moves retained with probability *P*(accept) = min(1, exp(−(Δ*Risk*_mut_ − Δ*Ris*k_current_)/*T*)) at exploration temperature *T*. This formulation reduced dependence on purely greedy updates and improved traversal of the local design landscape under each mutation budget. To quantify how search‐space size influenced optimization, we compared mutation budgets across independent runs and evaluated their global effect on search capacity using the Kruskal–Wallis test.

### 4.22 Multi‐Objective Pareto Analysis and Consensus Modelling

A comprehensive multi‐objective design landscape was constructed from sampled evolutionary trajectories and benchmarked against matched random mutants and deterministic greedy baselines. Final candidate mutations were not selected on the basis of aggregation suppression alone, but were interpreted jointly across aggregation‐risk reduction, structural cost, and evolutionary plausibility. To preserve this trade‐off structure, multi‐objective quality was quantified by computing a Pareto dominance count for each variant, defined as the total number of alternative sequences it strictly outperformed across all three optimization parameters. A low‐cost safe‐harbor regime was defined to isolate sequences achieving absolute aggregation suppression within strict structural constraints. Enrichment of evolutionary designs within this safe‐harbor region was assessed utilizing Fisher's exact tests, with OR adjusted by the Haldane–Anscombe correction to account for zero‐count contingency tables. Recurrent structural determinants of aggregation suppression were identified by isolating the top‐performing variants from each optimization run and calculating the per‐residue selection probability, with uncertainty bounded by 95% Wilson score intervals. To determine whether the observed mutational consensus reflected biophysical selection pressure rather than stochastic exploration, a null baseline was generated using unoptimized random‐walk sequence simulations. Statistical enrichment of recurrent design sites over this null background was evaluated using one‐sided Fisher's exact tests, with multiple comparisons controlled by the Benjamini–Hochberg FDR procedure.

### 4.23 Efficacy Retention Under Perturbation Stress Tests

The functional resilience of the computationally designed suppressors was evaluated utilizing in silico perturbation stress tests applied during the inference. Input representations for the top consensus variants and baseline controls were repeatedly perturbed with independent Gaussian coordinate jitter, continuous node feature noise, and stochastic graph edge dropout. Structural robustness was quantified using an efficacy retention score, defined as the empirical probability that a variant maintained effective aggregation suppression across perturbation iterations *ϵ*, expressed as Retention (*v*) = *P*(Δ*Risk* (*v*;*ϵ*) < *−δ*), where *δ* denotes a predefined minimum suppression margin used to prevent classification by marginal or saturated effects. Retention efficacy was compared between designed sequences and random baselines using two‐sided Mann–Whitney *U*‐test and Cliff's delta effect sizes.

## Author Contributions

J.S.L., W.X.W.L., and C.S.N. conceived the idea and designed the algorithm. W.X.W.L. and J.S.L. performed all the computational analyses of machine learning and calculations. W.X.T., Y.S.L., and H.L. contributed to the experiments. H.X.L. provided support on the experimentation. C.S.N. acquired funding, supervised the study, and provided feedback. All authors contributed to writing the paper and making the figures.

## Conflicts of Interest

The authors declare no conflicts of interest.

## Supporting information




**Supporting File 1**: advs76118‐sup‐0001‐SuppMat.pdf.

**Supporting File 2**: advs76118‐sup‐0001‐Data.zip.

## Data Availability

The data that support the findings of this study are available in the supplementary material of this article. Other training datasets are publicly available [64, 65]. The analysis code for this study is publicly available via GitHub at https://github.com/ngchenseng/SKALE2.0‐ALS‐aggregation‐GDL.git
