# Supplementary material for: A Phase‐Resolved Geometric Deep Learning Framework Maps Structural Determinants of Disease‐Associated Protein Aggregation and Guides Suppressor Design
Source: Adv Sci (Weinh). 2026 Jun 17:e76118. Online ahead of print. doi: 10.1002/advs.76118 (PMC13336538; doi:10.1002/advs.76118)
Supplement: Supplementary file 1 — Supporting File 1: advs76118‐sup‐0001‐SuppMat.pdf. [file ADVS-9999-e76118-s001.pdf]

Supplemental information

A phase-resolved geometric deep learning framework maps structural determinants of disease-associated protein aggregation and guides suppressor design

Jia Shen Sio, Wei Xuan Wilson Loo, Yan Shan Loo, Wen Xin Tan, Hui Xuan Lim, Huitao Liu, Chen Seng Ng

This file contains ten supplemental items:

Figure S1-S9

Supplemental Table 1

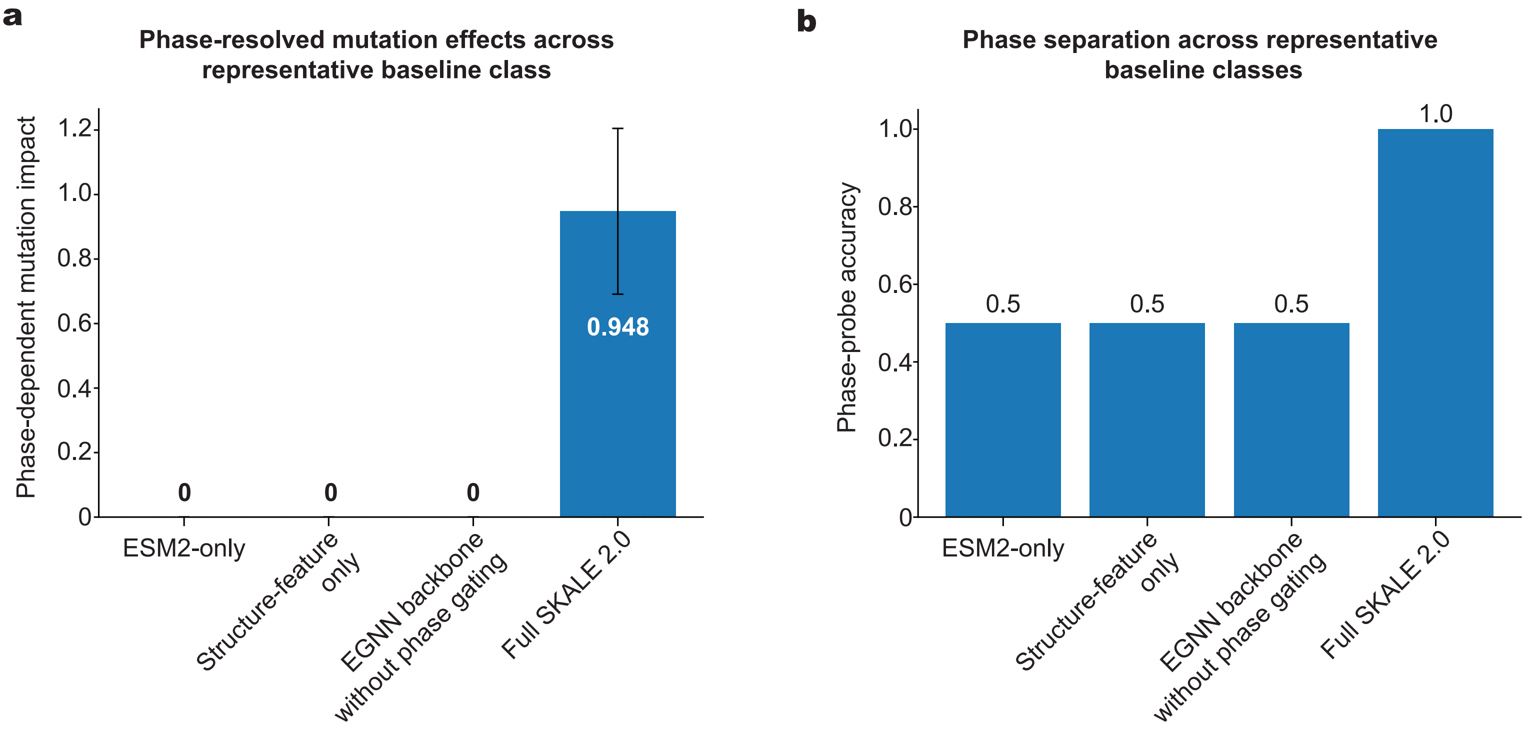

c

Model	Model class	Input representation	WT-mutant pairing	Phase aware	Phase modulation index	Phase modulation s.e.m	Mean modulation impact in nucleation	Mean modulation impact in elongation	Phase-probe accuracy	Phase silhouette score
ESM2-only	Protein language model	Mean pooled ESM2 embeddings only	No	No	0	0	0.113	0.113	0.5	0
Structure-feature only	AlphaFold-derived feature baseline	Mean pooled SASA, hydrogen bond, and fluctuation features	No	No	0	0	1.022	1.022	0.5	0
EGNN backbone without phase gating	Structure based deep learning baseline	Pooled EGNN latent representation before phase gating	No	No	0	0	2.384	2.384	0.5	0
Full SKALE 2.0	Phase-aware geometric deep learning	Phase-conditioned EGNN latent representations	Yes	Yes	0.948	0.257	3.426	2.478	1.0	0.345

Benchmark metrics quantify phase-dependent mutation modulation and phase separability across representative baseline classes. s.e.m. denotes standard error of the mean.

Fig. S1. Benchmarking SKALE 2.0 against representative baseline classes.

(**a**) Phase-resolved mutation effects across a protein language model baseline built from mean-pooled ESM2 embeddings, an AlphaFold-derived feature baseline, an EGNN backbone without phase gating, and full SKALE 2.0, quantified by the phase modulation index defined as the absolute difference in WT-to-mutant latent displacement between nucleation and elongation, with error bars denoting s.e.m. across WT-to-mutant pairs. (**b**) Phase-separation across the same baseline classes, evaluated by phase-probe accuracy for discrimination between nucleation and elongation embeddings. (**c**) Benchmark summary across all evaluated models, including model class, input representation, WT-mutant pairing status, phase awareness, phase modulation index, phase modulation s.e.m., mean mutation impact in nucleation, mean mutation impact in elongation, phase-probe accuracy, and phase silhouette score.

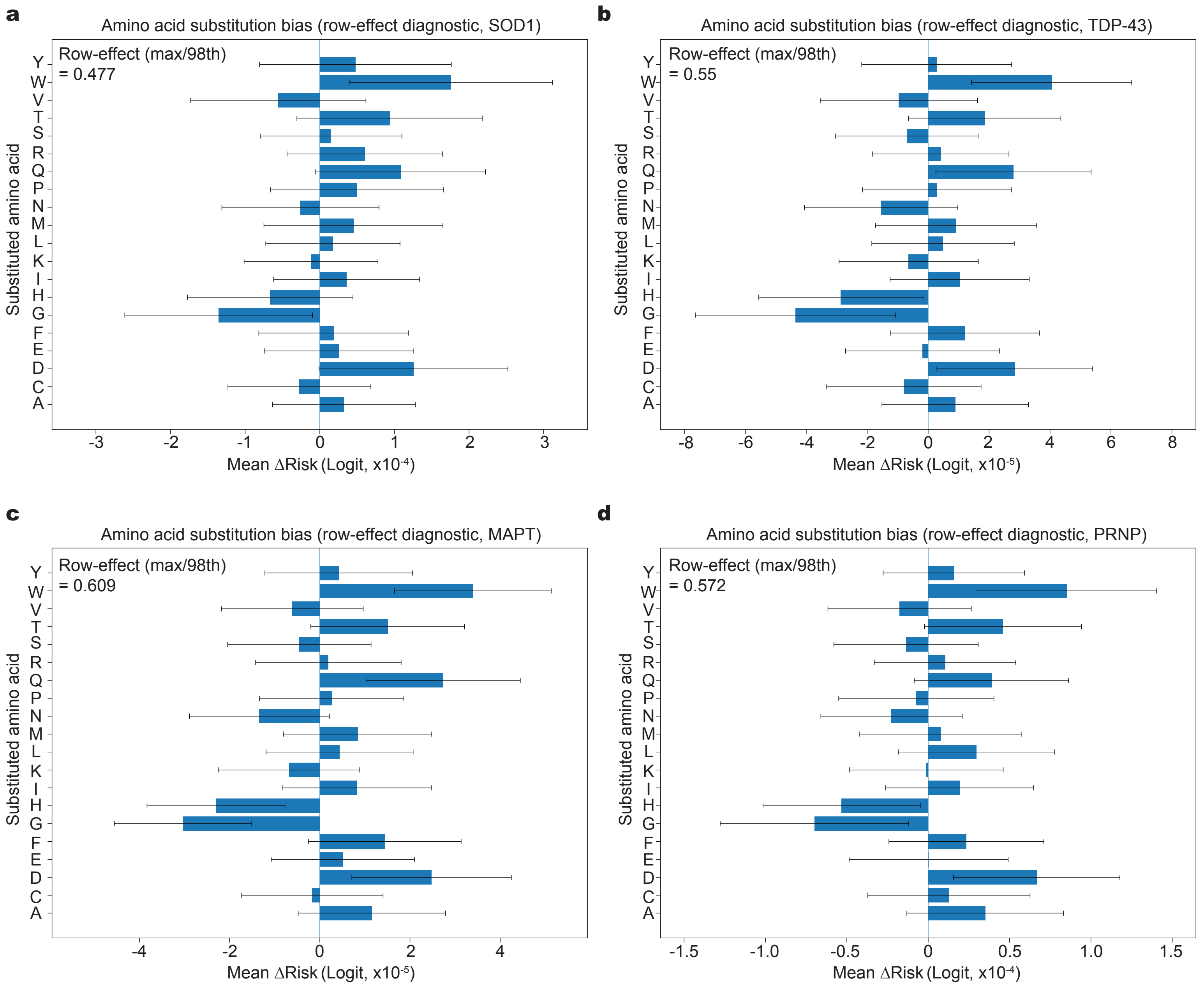

Fig. S2. Amino acid substitution bias diagnostics across structurally diverse proteins. Mean predicted aggregation risk shift ($\Delta Risk_{logit}$) associated with each introduced amino acid across all residue positions in the elongation-phase saturation mutagenesis atlases, with horizontal bars denoting the row mean and grey whiskers indicating the standard deviation across sequence positions. The vertical reference line marks zero mean shift. Inset report the row-effect summary statistic, defined as the maximum absolute row mean divided by the 98th percentile of the absolute risk shift ($|\Delta Risk_{logit}|$) for the corresponding atlas. Panels show substitution-bias diagnostics for (**a**) SOD1, (**b**) TDP-43, (**c**) MAPT, and (**d**) PRNP.

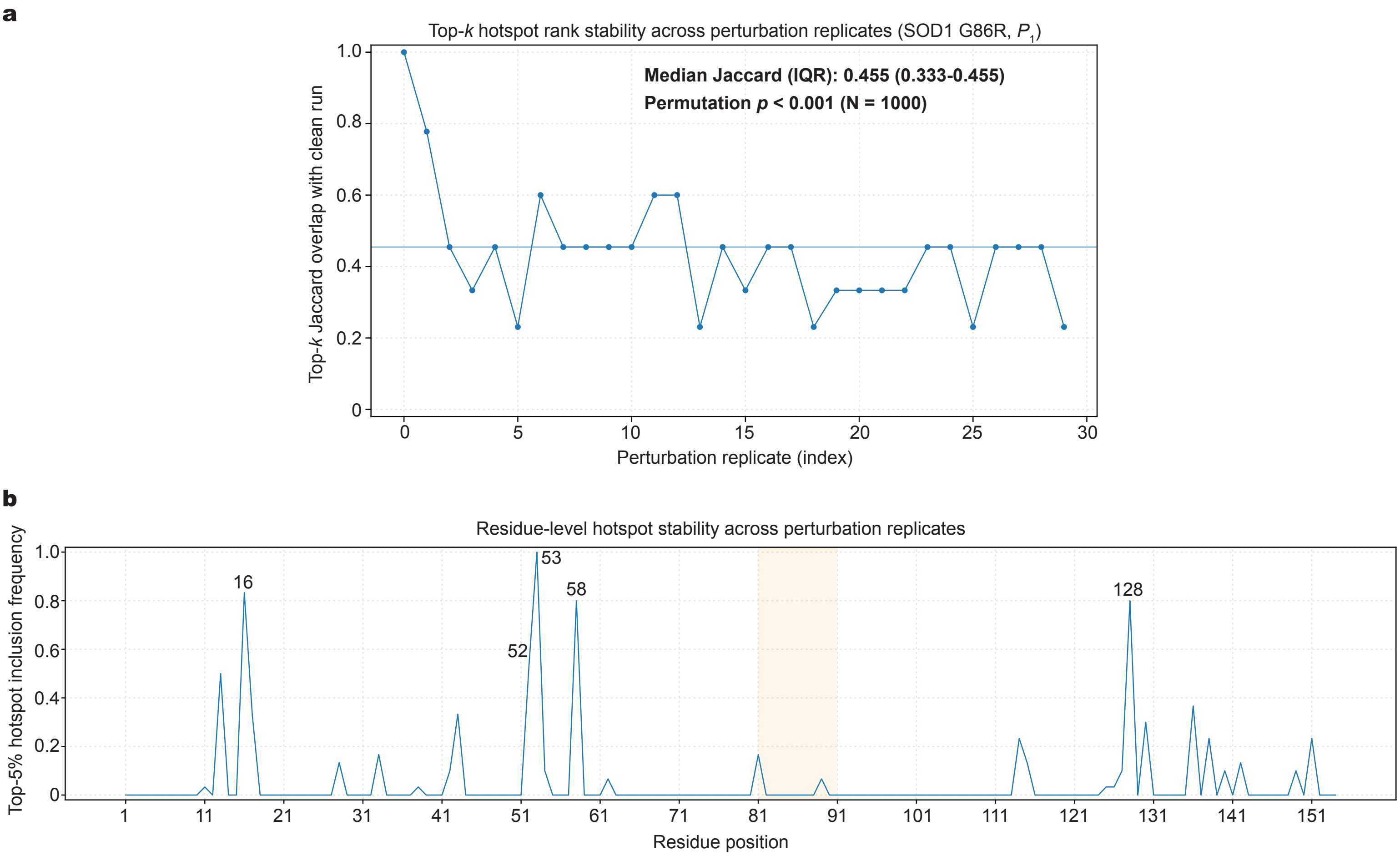

Fig. S3. Hotspot rank stability under perturbation replicates for SOD1 G86R under elongation-phase scoring.

(**a**) Consistency of the top-k hotspot set across perturbation replicates, quantified as the Jaccard overlap between the clean run and each perturbed replicate after ranking residues by sensitivity and selecting the top 5% ($k = \lceil 0.05 \cdot L \rceil$). The annotation reports the median overlap and interquartile range across replicates, together with a one-sided permutation test (N = 1,000) against a size-matched random-selection null. (**b**) Residue-wise inclusion frequency within the top-5% hotspot set across perturbation replicates, yielding a consensus stability profile, with labeled peaks marking residues most frequently retained among top-ranked hotspots. The shaded region denotes the mutation-centered window (±5 residues) shown for contextual reference.

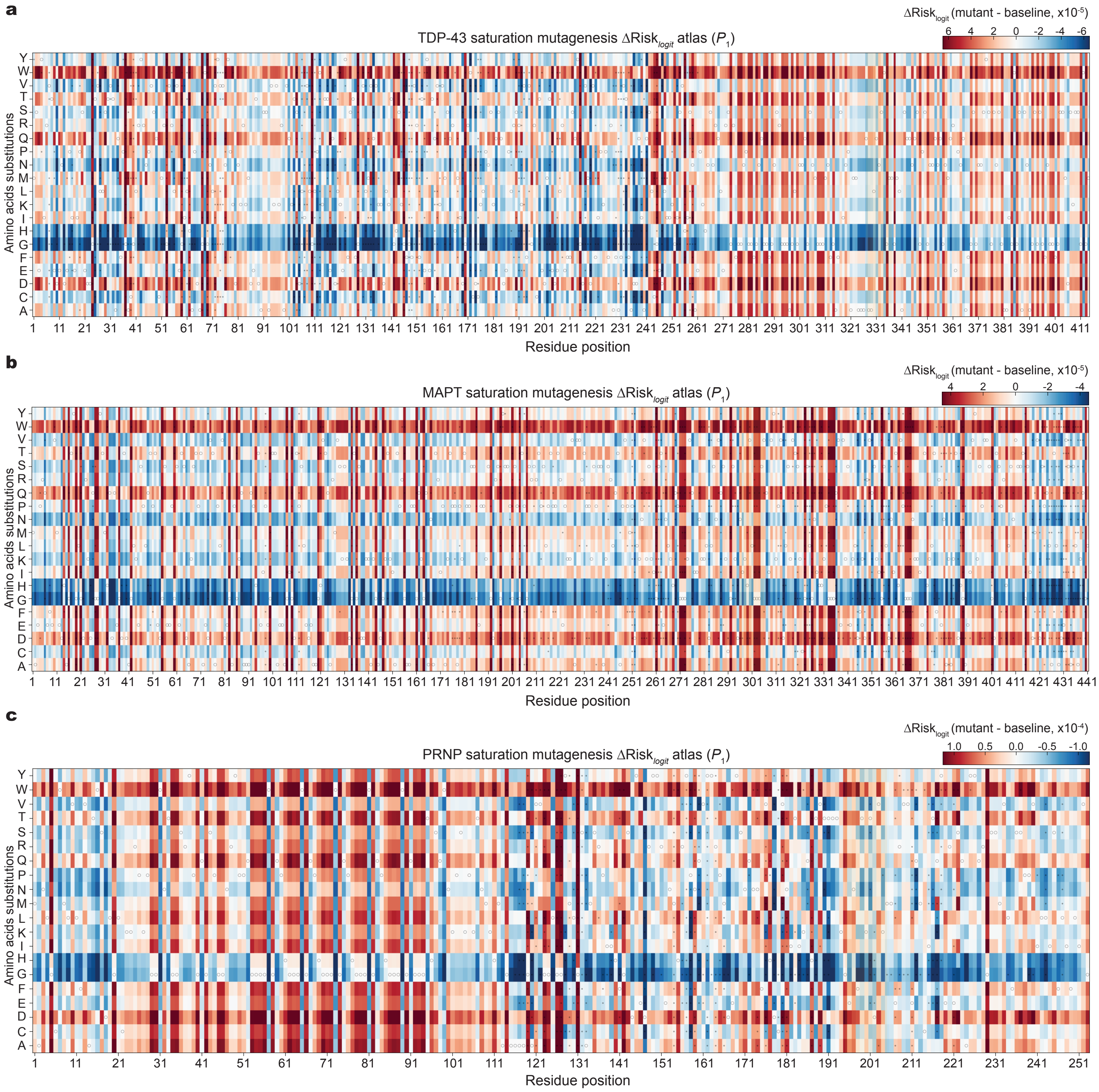

Fig. S4. Phase-conditioned saturation mutagenesis atlases across additional disease proteins. (**a-c**) Phase-conditioned single-amino acid substitution atlases for (**a**) TDP-43 S332N, (**b**) MAPT P301L, and (**c**) PRNP E200K under elongation phase scoring (P_1), with ΔRisk$_{\text{logit}}$ defined as the predicted aggregation-risk logit of the substituted sequence minus that of the parent sequence. Each cell reports the median across K = 30 perturbation replicates, including an unperturbed forward pass and stochastic perturbations with Gaussian feature noise (σ = 0.05), coordinate jitter (σ = 0.4 Å), and edge dropout (p = 0.08). Heatmaps are shown as row-centered atlases with per-amino acid row means subtracted and symmetric color limits defined by the CLIP_Q quantile of |values|. Hollow circles denote the WT residue identity at each position. Black stippling denotes high-uncertainty entries defined by median absolute deviation in the top decile across perturbation replicates. Replicate-wise Spearman ρ values relative to the clean replicate, together with P values where available, were exported for each target.

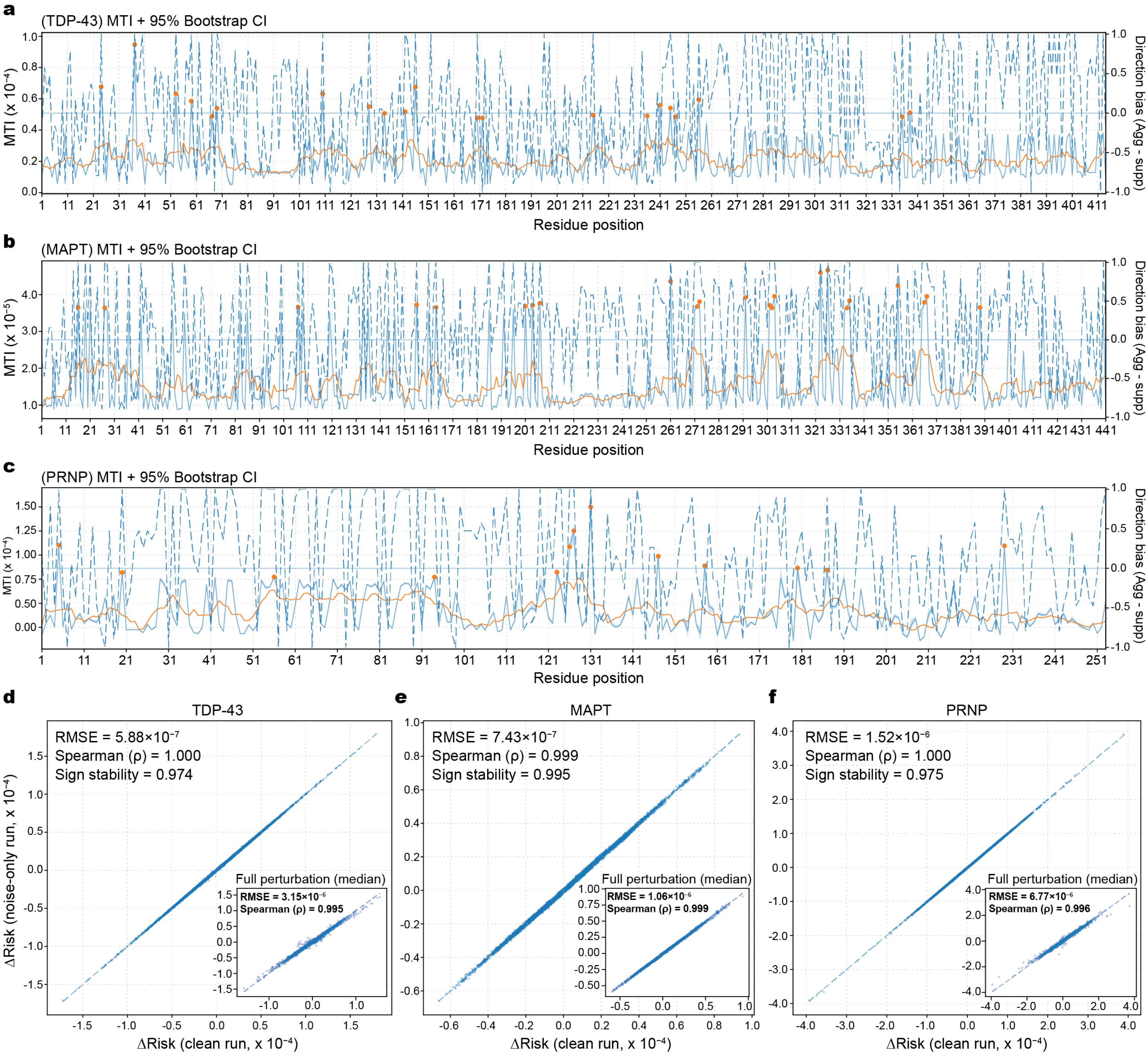

Fig. S5. Phase-conditioned mutational tolerance and robustness diagnostics across additional disease proteins.

(**a-c**) Residue-resolved MTI for (**a**) TDP-43 S332N, (**b**) MAPT P301L, and (**c**) PRNP E200K under elongation phase scoring (P_1), computed as the trimmed mean of $|\Delta\mathrm{Risk}_{\mathrm{logit}}|$ across the 19 non-synonymous substitutions at each position and reported as the median across K = 30 perturbation replicates. Shaded envelopes denote 95% CI obtained by block-bootstrap resampling of perturbation replicates (n = 500). The overlaid direction-bias track summarizes per-position imbalance between aggregation-leaning and suppressor-leaning substitutions, defined as $\mathrm{net}_{\mathrm{sign}} = \mathrm{frac}(\Delta\mathrm{Risk}_{\mathrm{logit}} > 0) - \mathrm{frac}(\Delta\mathrm{Risk}_{\mathrm{logit}} < 0)$. (**d-f**) Concordance between the clean unperturbed atlas against a noise-only replicate with Gaussian feature noise (σ = 0.05) for (**d**) TDP-43 S332N, (**e**) MAPT P301L, and (**f**) PRNP E200K, together with the median atlas obtained under full perturbation suite including Gaussian feature noise, coordinate jitter, and edge dropout.

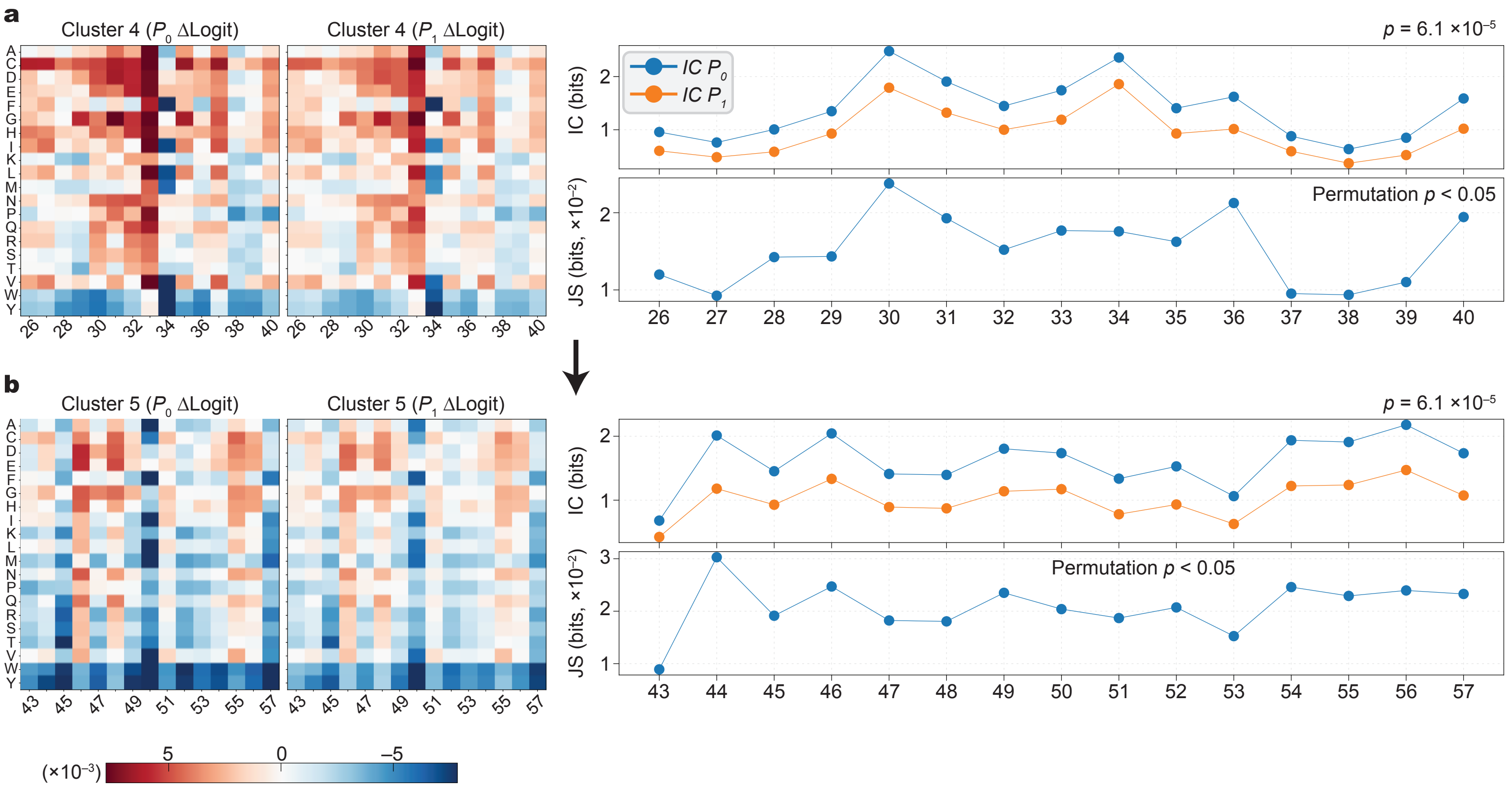

Fig. S6. Extended cluster-resolved phase-specific substitution landscapes in SOD1.

(**a**,**b**) Residue-resolved substitution landscapes for **a**, Cluster 4 and **b**, Cluster 5 in SOD1, with heatmaps showing the median predicted aggregation risk shift ($\Delta\text{Risk}_{\text{logit}}$) for all amino acid substitutions at each residue position under nucleation (P_0, top) and elongation (P_1, bottom) phase conditioning. Accompanying line plots quantify per-position amino acid preference strength as IC (bits) derived from softmax-normalized substitution distributions for P_0 and P_1. Phase divergence at each position is measured utilizing JS divergence (bits), with red stars denoting permutation-derived residue-level significance ($P < 0.05$), and annotated P values indicate two-sided paired Wilcoxon signed-rank tests across each cluster window. Residue numbering follows WT SOD1 sequence indexing. All substitution effects were computed using ESM2 re-embedding under phase-conditioned scoring.

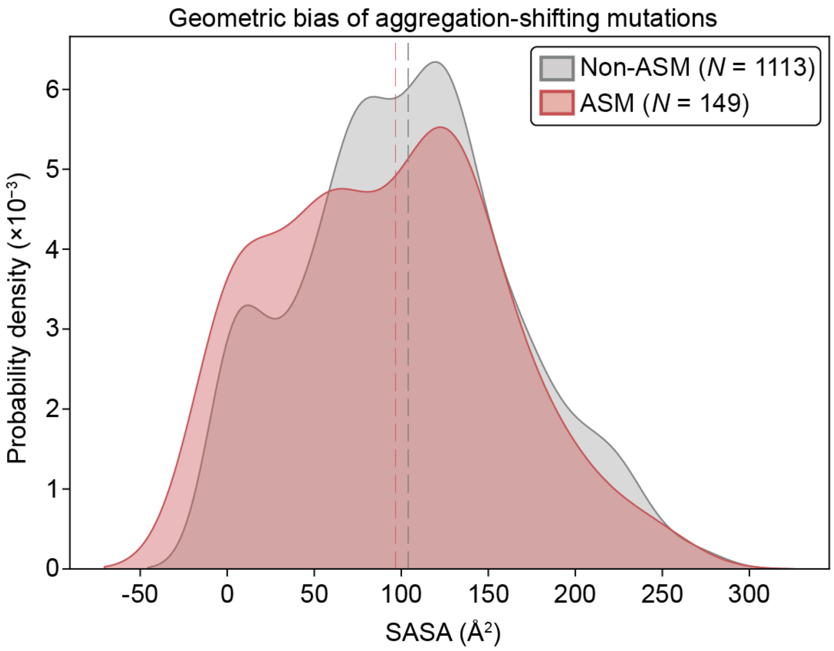

Fig. S7. Global geometric consensus of aggregation-sensitive mutations.

Kernel density distributions of SASA across ASM (Red, $N = 149$) and non-sensitive background residues (Non-ASM, grey, $N = 1113$) pooled across SOD1, TDP-43, MAPT, and PRNP. Dashed vertical lines denote the median SASA for each population.

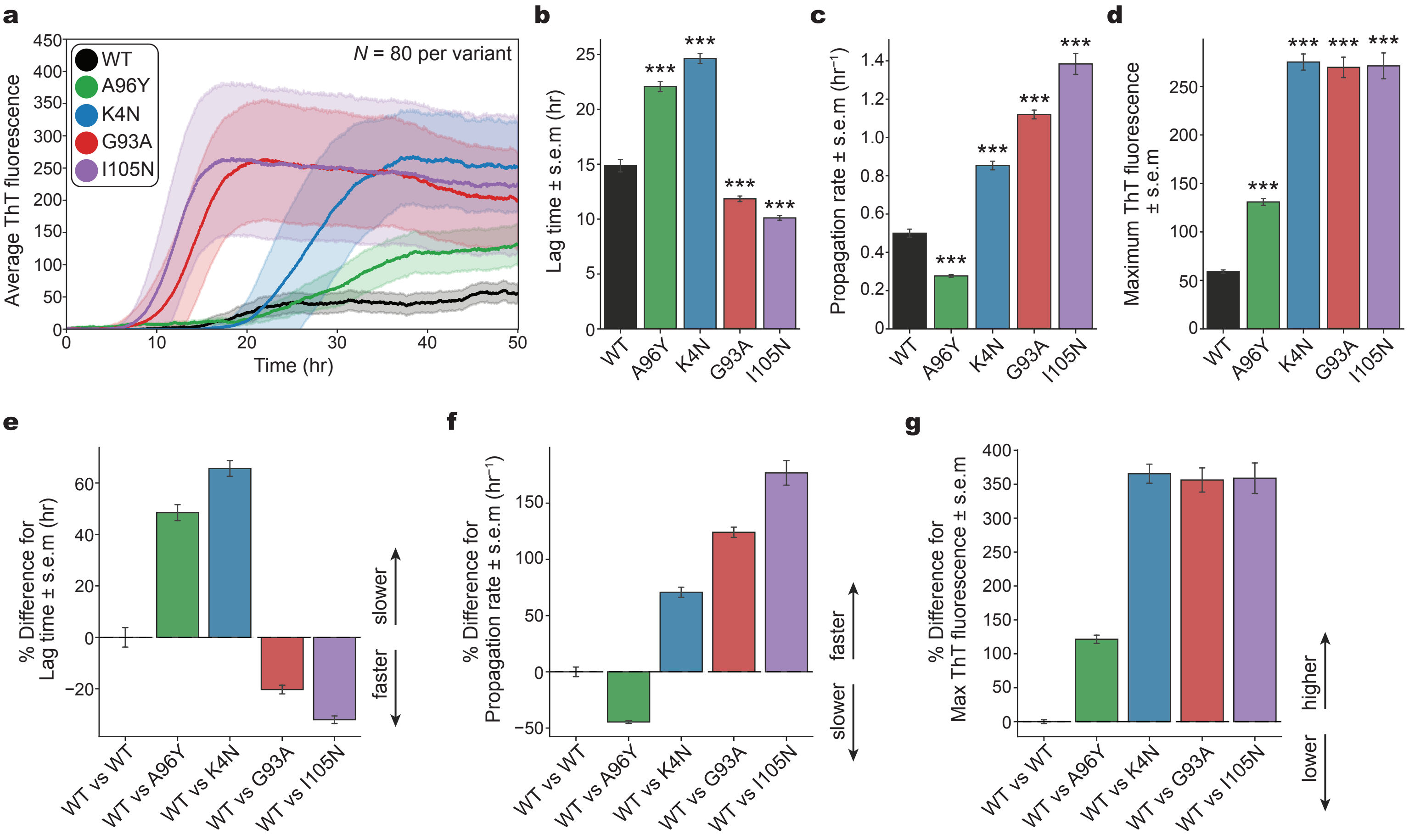

Fig. S8. Experimental validation of SKALE 2.0 predicted phase-specific aggregation kinetics in SOD1 (**a**) ThT fluorescence trajectories for recombinant SOD1 variants WT, A96Y, K4N, G93A, and I105N under aggregation-prone conditions, with markers represent the mean signal across a 50 h time course and shaded regions indicating ± s.d. (*N* = 80 independent replicates per variant). (**b-d**) Absolute kinetic parameters extracted from individual fluorescence trajectories by sigmoidal aggregation model: (**b**) lag time (t_{lag}), (**c**) apparent propagation rate (k_{app}), and (**d**) maximum ThT fluorescence at the plateau phase. (**e-g**) Percentage differences in kinetic parameters relative to the WT baseline (indicated by the dashed zero-line) for (**e**) lag time, (**f**) propagation rate, and (**g**) maximum fluorescence. Directional arrows indicate the direction of change relative to WT. Data in bar graphs are presented as mean ± s.e.m. Statistical significance was determined using a two-sided Welch's unpaired *t*-test comparing each variant with WT; ***$p < 0.001$.

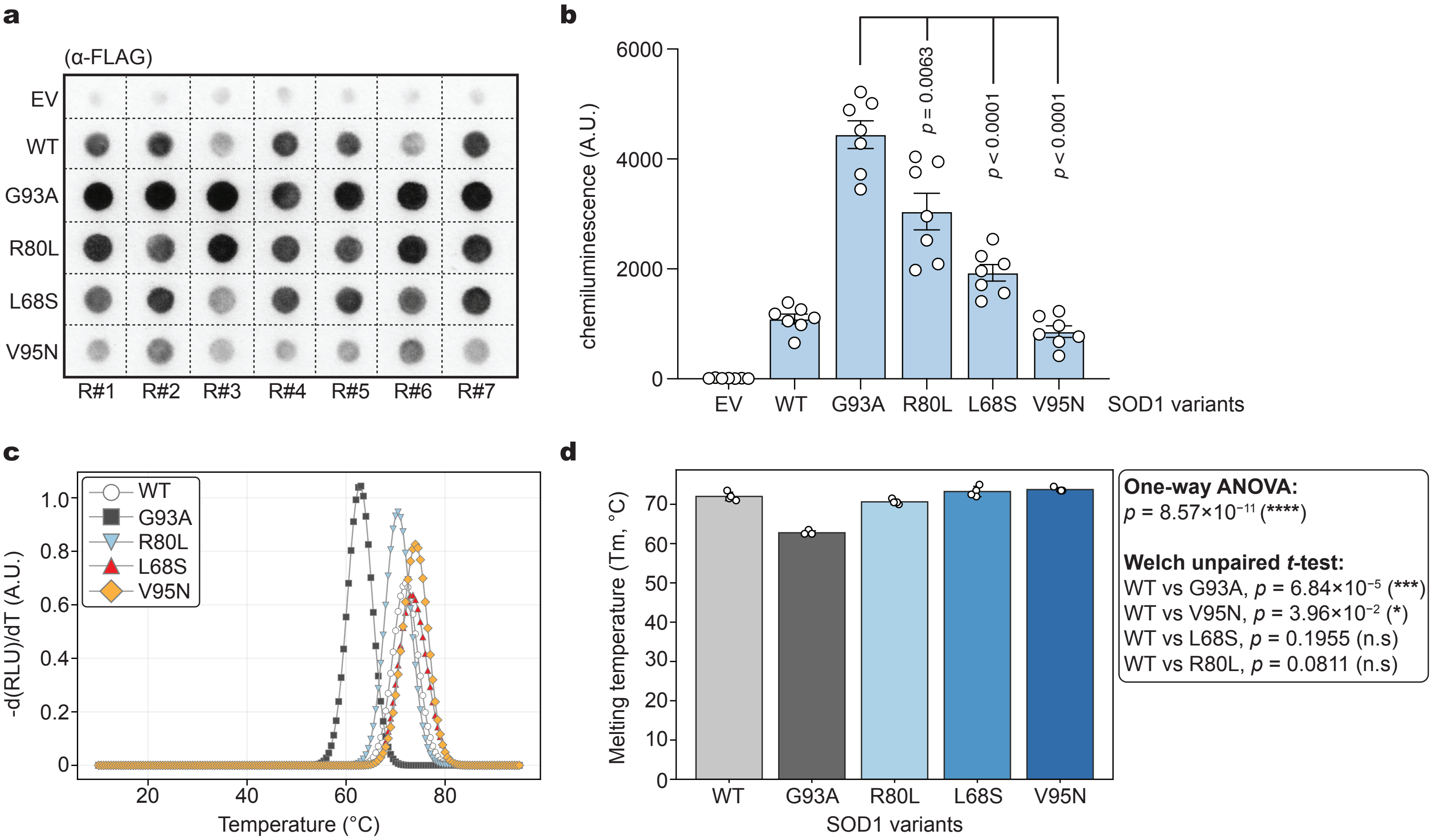

Fig. S9. Designed structural suppressors reduce intracellular SOD1 aggregation while preserving native thermodynamic stability.

(**a**) Filter-retardation analysis of detergent-insoluble SOD1 species in HEK293T cells expressing FLAG-tagged SOD1 variants, including empty vector (EV), WT, G93A, and the computationally nominated variants R80L, L68S and V95N. Cell lysates were filtered through cellulose acetate membranes and immunoblotted with anti-FLAG antibody. Seven independent biological replicates are shown (R#1–R#7). (**b**) Quantification of filter-retardation chemiluminescence signals from panel **a**. Individual measurements are shown as points and bars represent mean aggregate-retention signal. Statistical significance was determined using two-sided Welch's unpaired *t*-tests comparing each variant to G93A. (**c**) Derivative melting curves from SYPRO Orange thermal shift assays of purified recombinant SOD1 proteins, with unfolding transitions represented as the first derivative of fluorescence with respect to temperature, −d(RFU)/dT. Curves represent the replicate-averaged profiles for WT, G93A, R80L, L68S and V95N. (**d**) Melting temperatures (T_m) derived from the peak maxima of the derivative melt curves in panel **c**. Bars represent mean values and points showing individual replicates. Statistical analysis was performed using one-way ANOVA followed by Welch's two-sided unpaired *t*-tests for selected pairwise comparisons.

Supplementary Table 1. Structural cohort and supervision coverage used for SKALE 2.0

Protein family	Entry	Status	Paired WT → mutant set	Binary aggregation label	Role in model training
SOD1	WT	Wild type	SOD1 WT → G86R	No	Structural encoder, WT reference, kinetics supervision
SOD1	G86R	Mutant	SOD1 WT → G86R	No	Paired mutant structural input
TDP-43	WT	Wild type	TDP-43 WT → S332N	No	Structural encoder, WT reference, kinetics supervision
TDP-43	S332N	Mutant	TDP-43 WT → S332N	No	Paired mutant structural input
MAPT	WT	Wild type	MAPT WT → P301L	No	Structural encoder, WT reference
MAPT	P301L	Mutant	MAPT WT → P301L	No	Paired mutant structural input
PRNP	WT	Wild type	PRNP WT → E200K; MAPT WT → G127V	No	Structural encoder, WT reference
PRNP	E200K	Mutant	PRNP WT → E200K	No	Paired mutant structural input
PRNP	G127V	Mutant	MAPT WT → G127V	No	Paired mutant structural input

Note. The structural cohort comprised four protein families and nine total structure entries, including four WT references and five matched mutant structures, which yielded five WT to mutant Siamese pairs. Binary aggregation labels were not supplied in the present training manifest, so the conditional aggregation-risk head was not directly supervised.
